# Supplementary material for: In Vitro/In Vivo Translation of Synergistic Combination of MDM2 and MEK Inhibitors in Melanoma Using PBPK/PD Modelling: Part III
Source: Int J Mol Sci. 2023 Jan 23;24(3):2239. doi: 10.3390/ijms24032239 (PMC9917191; doi:10.3390/ijms24032239)
Supplement: Supplementary file 1 [file ijms-24-02239-s001.zip › ijms-2133015-supplementary.pdf]

Article Supplementary Materials

In Vitro/In Vivo translation of synergistic combination of MDM2 and MEK inhibitors in melanoma using PBPK/PD modelling: Part III

Jakub Witkowski, Sebastian Polak, Dariusz Pawelec and Zbigniew Rogulski

Table S1. Comparison of predicted vs observed $AUC_{0\text{-}inf}$ for siremadlin. Population representative and total population derived $AUC_{0\text{-}inf}$ parameter was generated with mixed zero and first order absorption mechanism and presented as geometric mean.

Dose (mg)	Representative $AUC_{0\text{-}inf}$ predicted (nM × h)	Population $AUC_{0\text{-}inf}$ predicted (nM × h)	$AUC_{0\text{-}inf}$ observed (nM × h)	Representative AUC predicted/ observed	Population AUC predicted/ observed
1	173.95	219.61	241.80	0.72	0.91
2	348.05	271.02	304.46	1.14	0.89
4	986.64	702.02	387.64	2.55	1.81
7.5	1644.47	1166.37	1076.86	1.53	1.08
12.5	3161.51	3936.16	2670.28	1.18	1.47
15	2492.59	2207.41	2343.49	1.06	0.94
20	3986.24	2930.14	4122.18	0.97	0.71
25	4965.94	3278.73	4803.12	1.03	0.68
50	10102.76	7234.90	14455.63	0.70	0.50
100	22727.82	16360.37	25723.34	0.88	0.64
120	22702.19	17494.86	26576.94	0.85	0.66
150	27016.21	20116.37	42719.97	0.63	0.47
200	39736.94	28563.71	47271.75	0.84	0.60
250	48268.48	36905.39	74579.68	0.65	0.49
350	80713.36	56905.60	99211.21	0.81	0.57

Table S2. Comparison of predicted vs observed C_{max} for siremadlin. Population representative and total population derived C_{max} parameter was generated with mixed zero and first order absorption mechanism and presented as geometric mean.

Dose (mg)	Representative C_{max} predicted (nM)	Population C_{max} predicted (nM)	C_{max} observed (nM)	Representative C_{max} predicted/ observed	Population C_{max} predicted/ observed
1	8.49	10.40	14.22	0.60	0.73
2	16.94	13.36	21.61	0.78	0.62
4	48.09	35.27	31.69	1.52	1.11
7.5	80.10	58.58	70.22	1.14	0.83
12.5	154.14	186.60	212.46	0.73	0.88
15	121.45	110.89	164.74	0.74	0.67
20	194.09	147.70	269.17	0.72	0.55
25	242.37	166.34	422.57	0.57	0.39
50	493.11	363.55	840.82	0.59	0.43
100	1109.85	821.79	1194.25	0.93	0.69
120	1107.72	884.61	2299.74	0.48	0.38
150	1314.86	1009.07	2600.42	0.51	0.39
200	1939.33	1449.07	2104.39	0.92	0.69
250	2353.38	1852.15	3629.21	0.65	0.51
350	3928.43	2887.66	4066.91	0.97	0.71

Table S3. Differences in PBPK model parameters with and without PK interaction at absorption level.

Drug/ Parameter	ka (1/h) {%CV}	tlag (h) {%CV}	fa (%) {%CV}
siremadlin	1.2 {30}	0.9 {30}	0.83 {30}
siremadlin (PK interaction)	2.2 {30}	0.9 {30}	0.934 {30}
trametinib	0.6 {40}	0.35 {40}	0.72 {40}
trametinib (PK interaction)	0.252 {40}	0	0.608 {40}

ka: absorption rate constant. *tlag*: lag time. *fa*: fraction of dose absorbed.

Table S4. Parameters of the clinical PBPK model for siremadlin.

Model Section	Parameter (Units)	Value {%CV}	Source/Reference/Comments
**Physiochemical properties and blood binding**	Molecular Weight (g/mol)	555.41	-
	logP	2.99	*In vitro* determined - Unpublished Adamed Pharma data (value similar to reported 2.90 [1])
	Compound Type	Monoprotic Base	-
	pKa	1.69	*In vitro* determined - Unpublished Adamed Pharma data
	B/P	0.61	*In vitro* determined - Unpublished Adamed Pharma data (arithmetic mean from 2.5-10uM - range 0.49-0.70)
	fu plasma	0.18313	*In vitro* determined - Unpublished Adamed Pharma data (arithmetic mean from 2.5-10uM range after 18h of incubation which was needed to reach equilibrium – range 0.1601-0.1966)
**Absorption**	Absorption model	First-Order	-
	fa	0.83 {30}	Optimized
	ka (1/h)	1.2 {30}	Optimized
	Lag time (h)	0.9 {30}	Optimized
	Caco-2 Apical pH: Basolateral pH – 6.5:7.4 (10^{-6} cm/s)	6.4	In vitro determined - Unpublished Adamed Pharma data

Model Section	Parameter (Units)	Value {%CV}	Source/Reference/Comments
	Caco-2 reference Papp (10^{-6} cm/s)	22.4	In vitro determined - Unpublished Adamed Pharma data
Distribution	Distribution model	Full PBPK	-
	Vss (L/kg)	1.617	Simcyp predicted (Method 3) – value similar to literature value [2] and allometrically scaled Vss from mice and rat data [3,4] (120L/74.21kg=1.617 L/kg)
	Smoothing function	Enabled	-
	Sub-Cellular Distribution model	Enabled	Only for Adipose and Bone tissues
	Olive oil:water partition as a surrogate for neutral lipid partition option	Disabled	-
	Kp Brain	0.094	Previously presented data [5]
	Kp Gut	6.3976	Previously presented data [5]
	Kp Heart	3.0107	Previously presented data [5]
	Kp Kidney	3.7123	Previously presented data [5]
	Kp Liver	5.0403	Previously presented data [5]
	Kp Lung	2.0733	Previously presented data [5]
	Kp Muscle	1.7894	Previously presented data [5]
	Kp Skin	2.8584	Previously presented data [5]
	Kp Spleen	2.2213	Previously presented data [5]
	Kp Scalar	0.11286	Optimized
Elimination	Clearance type	Human Hepatocytes	-

Model Section	Parameter (Units)	Value {%CV}	Source/Reference/Comments
	Hep intrinsic CL (mL/min/10^6 cells)	1.64 {27.88}	Optimized from range 1.08-1.64 (In vitro determined - Unpublished Adamed Pharma data)
	fu_inc	0.5986	Optimized from range 0.5986-0.7520 (In vitro determined - Unpublished Adamed Pharma data)
	Additional renal CL	1.8636 {30}	Back-calculated based on in vivo mouse and rat clearance (3.12127 and 0.23 L/h respectively with rat fu=0.160167) using free fraction corrected intercept (FCIM) method [6]
**Tumour**	Tumour model type	Permeability-limited tumour model	-
	Tumour blood flow (mL/min)	0.76 {30}	Simcyp predicted for solid tumours
	Tumour PS (mL/min/mL of tumour volume)	0.020963	Optimized
	Tumour P-gp efflux transporter CL_{int} (mL/min mL tumour)	209.67	Optimized
	fu_{EW}	0.28932	Optimized
	fu_{IC}	0.0033979	Optimized
**Interaction**	CYP1A2 Ki	25	In vitro determined - Unpublished Adamed Pharma data, fumic assumed the same as in hepatocytes: 0.5986
	CYP2C9 Ki (uM)	25	In vitro determined - Unpublished Adamed Pharma data, fumic assumed the same as in hepatocytes: 0.5986

Model Section	Parameter (Units)	Value {%CV}	Source/Reference/Comments
	CYP3A4 Ki (uM)	20.5	In vitro determined - Unpublished Adamed Pharma data, fumic assumed the same as in hepatocytes: 0.5986
	CYP1A2 Ind_max	21.9 {30}	In vitro determined - Unpublished Adamed Pharma data, fumic assumed the same as in hepatocytes: 0.5986
	CYP1A2 Ind_50 (uM)	59.67 {30}	Simcyp predicted
	CYP2B6 Ind_max	1.7 {30}	In vitro determined - Unpublished Adamed Pharma data, fumic assumed the same as in hepatocytes: 0.5986
	CYP3A4 Ind_max	19 {30}	In vitro determined - Unpublished Adamed Pharma data, fumic assumed the same as in hepatocytes: 0.5986
	CYP3A4 Ind_50 (uM)	10 {30}	Simcyp predicted
**Trial design**	Administration route	Oral	-
	Dose (mg)	120	-
	Dose interval τ (h)	168	-
	Condition	Fasted	-
	Simulation duration	5040h	-
**Population**	Population	Sim-Cancer	Simcyp predicted
	Age range	20-80	Similar to reported 18-80 [7]
	Proportion of females	0.44	[7]

B/P: blood to plasma partition ratio. *fu plasma:* fraction unbound in plasma. *fa*: fraction of dose absorbed. *ka*: absorption rate constant. *Vss*: volume of distribution at steady-state. *Kp*: tissue-to-plasma partition coefficient. Hep:

Hepatocytes. *CL*: clearance. *fu_inc*: fraction of unbound drug in the *in vitro* system. Tumour *PS*: Passive permeability clearance between intra- and extracellular water of tumour. *Tumour P-gp efflux transporter* CL_{int}: *In vitro* transporter mediated intrinsic clearance in tumour. fu_{EW}: fraction unbound in the extracellular water of tumour. Fu_{IC}: fraction unbound in the intracellular water of tumour. *Ki*: concentration of compound that supports half maximal inhibition. *Fu_mic*: fraction of unbound drug in the in vitro microsomal incubation. *Ind_max*: maximal fold induction over vehicle. *Ind_50*: compound concentration that supports half maximal induction.

Table S5. Parameters of the clinical PBPK model for trametinib.

Model Section	Parameter (Units)	Value {%CV}	Source/Reference/Comments
**Physiochemical properties and blood binding**	Molecular Weight (g/mol)	615.39	-
	logP	4.10	*In vitro* determined - Unpublished Adamed Pharma data
	Compound Type	Monoprotic Base	-
	pKa	11.15	*In vitro* determined - Unpublished Adamed Pharma data
	B/P	0.56	*In vitro* determined - Unpublished Adamed Pharma data (range – 0.50-0.56)
	fu plasma	0.05	[8]
**Absorption**	Absorption model	First-Order	-
	fa	0.72 {40}	[8]
	ka (1/h)	0.6 {40}	Optimized
	Lag time (h)	0.35 {40}	Optimized
	Peff, man (10^{-4} cm/s)	1.07	[8]
**Distribution**	Distribution model	Full PBPK	-
	Vss (L/kg)	4.725	Simcyp predicted (Method 3): 4.725 – value in range of reported data 385-1836L with digitized mean patient weight of 81.479kg [9]
	Smoothing function	Enabled	-
	Sub-Cellular Distribution model	Enabled	Only for Adipose and Bone tissues

Model Section	Parameter (Units)	Value {%CV}	Source/Reference/Comments
	Olive oil:water partition as a surrogate for neutral lipid partition option	Disabled	-
	Kp Brain	0.1839	Previously presented data [5]
	Kp Gut	5.4756	Previously presented data [5]
	Kp Heart	1.3516	Previously presented data [5]
	Kp Kidney	3.2620	Previously presented data [5]
	Kp Liver	5.3955	Previously presented data [5]
	Kp Lung	1.3201	Previously presented data [5]
	Kp Muscle	1.0260	Previously presented data [5]
	Kp Skin	1.1427	Previously presented data [5]
	Kp Spleen	2.6154	Previously presented data [5]
	Kp Scalar	430.91	Optimized
**Elimination**	Clearance type	I.V. clearance	-
	CL_{iv} (mL/min)	5.4 {40}	[8]
**Tumour**	Tumour model type	Permeability-limited tumour model	-
	Tumour blood flow (mL/min)	0.35 {30}	Set to value observed in human melanoma xenografts [10–12]

Model Section	Parameter (Units)	Value {%CV}	Source/Reference/Comments
	Tumour PS (mL/min/mL of tumour volume)	0.0081	Optimized
	Tumour P-gp efflux transporter CL_{int} (mL/min mL tumour)	27.5	Optimized
	fu_{EW}	0.053175	Optimized
	fu_{IC}	0.78909	Optimized
**Interaction**	CYP2C9 Ki (uM)	2.1	Data from [8] fumic assumed the same as in microsomes: 0.58
	CYP3A4 Ki (uM)	3.2	Data from [8] fumic assumed the same as in microsomes: 0.58
	CYP3A4 Indmax	37.3 {30}	Data from [8] fumic assumed the same as in microsomes: 0.58
	CYP3A4 Ind50 (uM)	2.7 {30}	Simcyp predicted
**Trial design**	Administration route	Oral	-
	Dose (mg)	2	-
	Dose interval τ (h)	24	-
	Condition	Fasted	-
	Simulation duration	5040h	-

Model Section	Parameter (Units)	Value {%CV}	Source/Reference/Comments
**Population**	Population	Sim-Cancer	Simcyp predicted
	Age range	20-80	Similar to reported 23-85 [13]
	Proportion of females	0.44	[13]

B/P: blood to plasma partition ratio. *fu plasma:* fraction unbound in plasma. *fa*: fraction of dose absorbed. *ka*: absorption rate constant. *Peff, man*: Human jejunum effective permability. *Vss*: volume of distribution at steady-state. *Kp*: tissue-to-plasma partition coefficient. Hep: Hepatocytes. *CL*: clearance. *fu_inc*: fraction of unbound drug in the *in vitro* system. Tumour *PS*: Passive permeability clearance between intra- and extracellular water of tumour. *Tumour P-gp efflux transporter* CL_{int}: *In vitro* transporter mediated intrinsic clearance in tumour. fu_{EW}: fraction unbound in the extracellular water of tumour. Fu_{IC}: fraction unbound in the intracellular water of tumour. *Ki*: concentration of compound that supports half maximal inhibition. *Fu_mic*: fraction of unbound drug in the in vitro microsomal incubation. *Ind_max*: maximal fold induction over vehicle. *Ind_50*: compound concentration that supports half maximal induction.

Table S6. Parameters of the PD (TGI) models for single administration of siremadlin, trametinib. Models outcomes are depicted in Figures 6-9 and S22-S44.

Compound/ Parameter	Description	Siremadlin {%CV}**		Trametinib {%CV}**	
Dose	Compound dose (mg)	12.5/25/50/100/200/250/350 (regimen 1A)<br>120/150/200 (regimen 1B)<br>1/2/4/7.5/15/20 (regimen 2A)<br>15/20/25 (regimen 2C)			
SLD0	initial tumour size (cm)	12.226/9.655/9.823/11.082/9.654/9.325/11.012 (regimen 1A)<br>9.179/8.592/8.145 (regimen 1B)<br>8.478/8.319/11.900/10.526/7.987/9.539 (regimen 2A)<br>9.152/9.781/8.862 (regimen 2C)	{74.3}<br>{74.3}<br>{74.3}<br>{74.3}	6.4	{40.9}
kgh	initial Tumour growth rate (1/h)	0.00028/ 0.0000261	{30.7}	0.00028/ 0.0000261	{30}
fs	tumour sensitive fraction (%)	0.0321	{636}	0.191	{100}
lambda	Resistance factor	132	{10}	94.3	{10}

tau	Effect delay (h)	558	{100}	2.5	{10}
TSCs	Tumour Static concentration (nM)	1.015*	{10}	0.258/0.177	{10}
Mean Relative Error (%)***	-	14.73/20.06/2.76/16.77/14.24/4.25/7.65 (regimen 1A)<br>17.08/8.97/8.38 (regimen 1B)<br>9.51/5.65/6.70/4.48/7.97/15.13 (regimen 2A)<br>2.24/5.45/2.26 (regimen 2C)		0.10/0.55	

*TSCs value was converted from ng/mL to nM (0.564 ng/mL/ 0.55541 ng/nM).
** CV% was calculated according to equation: $\sqrt{e^{\omega^2}-1}$ [14].
***calculated for patient representatives (n = 1).

Table S7. Relationships between TGI model parameters for siremadlin and trametinib combination.

**Parameter/ Compound**	**siremadlin (HDM)**	**trametinib (TRA)**	**siremadlin+ trametinib combination**
SLD0	As median of resimulated data	As median in literature	As in trametinib arm (6.4) for Cases A, B, C<br>4.4 for Case D
kgh	2 subpopulations<br>0.00028/0.0000261	2 subpopulations<br>0.00028/0.0000261	2 subpopulations<br>0.00028 for case A, C, D<br>0.0000261 for case B
AUC ratio*	2.744-1.834*TRA dose-0.017*HDM dose+0.019*HDM dose * TRA dose	-0.000016*TRA dose * (HDM dose)^2+0.00057* (HDM dose)^2 *(TRA dose)^2-0.006*(HDM dose)$^{1.5}$*(TRA dose)$^{1.5}$ +1.36	-
TSCs	1.015	For 2 subpopulations<br>0.256/0.177	Case A: HDM TSCs – TRA TSCs<br>Case B: (TSCs_HDM - TSCs_TRA)/gamma<br>Case C: (TSCs_HDM/AUCratioHDM201 - TSCs_TRA/AUCratiotrametinib)<br>Case D: (TSCs_HDM/AUCratioHDM201 - TSCs_TRA/AUCratiotrametinib)/gamma
lambda	Estimated (x)	Estimated (y)	Max (x;y)

tau	Estimated (x)	Estimated (y)	$\lvert$HDM tau-TRA tau$\rvert$
fs	Estimated (x)	Estimated (y)	Case A, B, D: x+y<br>Case C: x * (HDM IIV: 1.93)
gamma	-	-	From *in vitro* synergy package analysis [15]

* Relationship between parameters derived from animals [5], using doses in mg/kg assuming 70 kg patient (see Table S8).

Table S8. Calculations of the AUC ratio parameters for siremadlin and trametinib combination at clinically tested doses based on previously published universal model in animals (Table S7 and [5]).

siremadlin dose (mg)	trametinib dose (mg)	Estimated siremadlin AUC ratio	Estimated trametinib AUC ratio
1	2	2.0451	1.3602
2	2	2.0432	1.3600
4	2	2.0395	1.3594
7.5	2	2.0330	1.3580
12.5	2	2.0237	1.3555
15	2	2.0191	1.3540
20	2	2.0098	1.3508
25	2	2.0005	1.3472
50	2	1.9541	1.3252
100	2	1.8612	1.2690
120	2	1.8240	1.2438
150	2	1.7683	1.2040
200	2	1.6754	1.1345
250	2	1.5825	1.0630
350	2	1.3967	0.9204

Table S9. Parameters for simulations of tumour size in siremadlin + trametinib combination for Case A. Simulation outcomes are depicted in Figure 10.

Parameter	siremadlin only<br>Case A	trametinib only<br>Case A	Case 1a	Case 2a	Case 3a	Case 4a
SLD0	6.4	6.4	6.4	6.4	6.4	6.4
kgh	0.00028	0.00028	0.00028	0.00028	0.00028	0.00028
fs	0.0321	0.191	0.2231	0.2231	0.2231	0.2231
lambda	132	94.3	132	132	132	132
tau	558	2.5	555.5	555.5	555.5	555.5
Total TSCs	1.015	0.258	0.7575	0.6152	0.3493	0.2837
AUCR HDM	-	-	-	-	1.8240	1.8240
AUCR TRA	-	-	-	-	1.2438	1.2438
gamma	-	-	-	1.2312	-	1.2312

blood flow*	0.35	0.35	0.35	0.35	0.35	0.35
PD DDI	no	no	no	yes	no	yes
PK DDI	no	no	no	no	yes	yes

*For simulations of combination in patients with melanoma tumour blood flow was adjusted to already measured blood perfusion in human melanoma xenografts [10–12].

Table S10. Parameters for simulations of tumour size in siremadlin + trametinib combination for Case B. Simulation outcomes are depicted in Figure 11.

Parameter	siremadlin only Case B	trametinib only Case B	Case 1b	Case 2b	Case 3b	Case 4b
SLD0	6.4	6.4	6.4	6.4	6.4	6.4
kgh	0.0000261	0.0000261	0.0000261	0.0000261	0.0000261	0.0000261
fs	0.0321	0.191	0.2231	0.2231	0.2231	0.2231
lambda	132	94.3	132	132	132	132
tau	558	2.5	555.5	555.5	555.5	555.5
Total TSCs	1.015	0.177	0.8385	0.6810	0.4144	0.3366
AUCR HDM	-	-	-	-	1.8240	1.8240
AUCR TRA	-	-	-	-	1.2438	1.2438
gamma	-	-	-	1.2312	-	1.2312
blood flow	0.35	0.35	0.35	0.35	0.35	0.35
PD DDI	no	no	no	yes	no	yes
PK DDI	no	no	no	no	yes	yes

Table S11. Parameters for simulations of tumour size in siremadlin + trametinib combination for Case C. Simulation outcomes are depicted in Figure 12.

Parameter	siremadlin only Case C	trametinib only Case C	Case 1c	Case 2c	Case 3c	Case 4c
SLD0	6.4	6.4	6.4	6.4	6.4	6.4
kgh	0.00028	0.00028	0.00028	0.00028	0.00028	0.00028
fs	0.0321	0.194	0.06195	0.06195	0.06195	0.06195
lambda	132	94.3	132	132	132	132
tau	558	2.5	555.5	555.5	555.5	555.5
Total TSCs	1.015	0.258	0.7575	0.6152	0.3493	0.2837
AUCR HDM	-	-	-	-	1.8240	1.8240
AUCR TRA	-	-	-	-	1.2438	1.2438
gamma	-	-	-	1.2312	-	1.2312
blood flow	0.35	0.35	0.35	0.35	0.35	0.35
PD DDI	no	no	no	yes	no	yes
PK DDI	no	no	no	no	yes	yes

Table S12. Parameters for simulations of tumour size in siremadlin + trametinib combination for Case D. Simulation outcomes are depicted in Figure 13.

Parameter	siremadlin only Case D	trametinib only Case D	Case 1d	Case 2d	Case 3d	Case 4d
SLD0	4.4	4.4	4.4	4.4	4.4	4.4
kgh	0.00028	0.00028	0.00028	0.00028	0.00028	0.00028
fs	0.0321	0.191	0.2231	0.2231	0.2231	0.2231
lambda	132	94.3	132	132	132	132
tau	558	2.5	555.5	555.5	555.5	555.5
Total TSCs	1.015	0.258	0.7575	0.6152	0.3493	0.2837
AUCR HDM	-	-	-	-	1.8240	1.8240
AUCR TRA	-	-	-	-	1.2438	1.2438
gamma	-	-	-	1.2312	-	1.2312
blood flow	0.35	0.35	0.35	0.35	0.35	0.35
PD DDI	no	no	no	yes	no	yes
PK DDI	no	no	no	no	yes	yes

Table S13. Parameters from [2] used for resimulation of siremadlin pharmacokinetics and pharmacodynamics in Simulx.

**Parameter**	**Value**	**omega**
Tk01_pop	1.11 (h)	0.07
ka2_pop	1 (1/h)	1.35
F1_pop	0.753	0.04
Tlag1_pop	0.688 (h)	0.05
Tlag2_pop	0.41 (h)	0.02
V_pop	0.12 (L)	0.333
CL_pop	0.00694 (L/h)	0.482
SLD0_pop	9.47 (cm)	0.663
kgh_pop	0.00028/0.0000261 (1/h)	0.3
fs_pop	0.0321	1.93
TSCs_pop	0.564 (ng/mL)	0.1
lambda_pop	132	0.1
tau_pop	558 (h)	0.836
a1_PD	0.111 (cm)	-
b1_PD	3.55 (%)	-
a1_PK	0.419 (ng/mL)	-
b1_PK	16.8 (%)	-

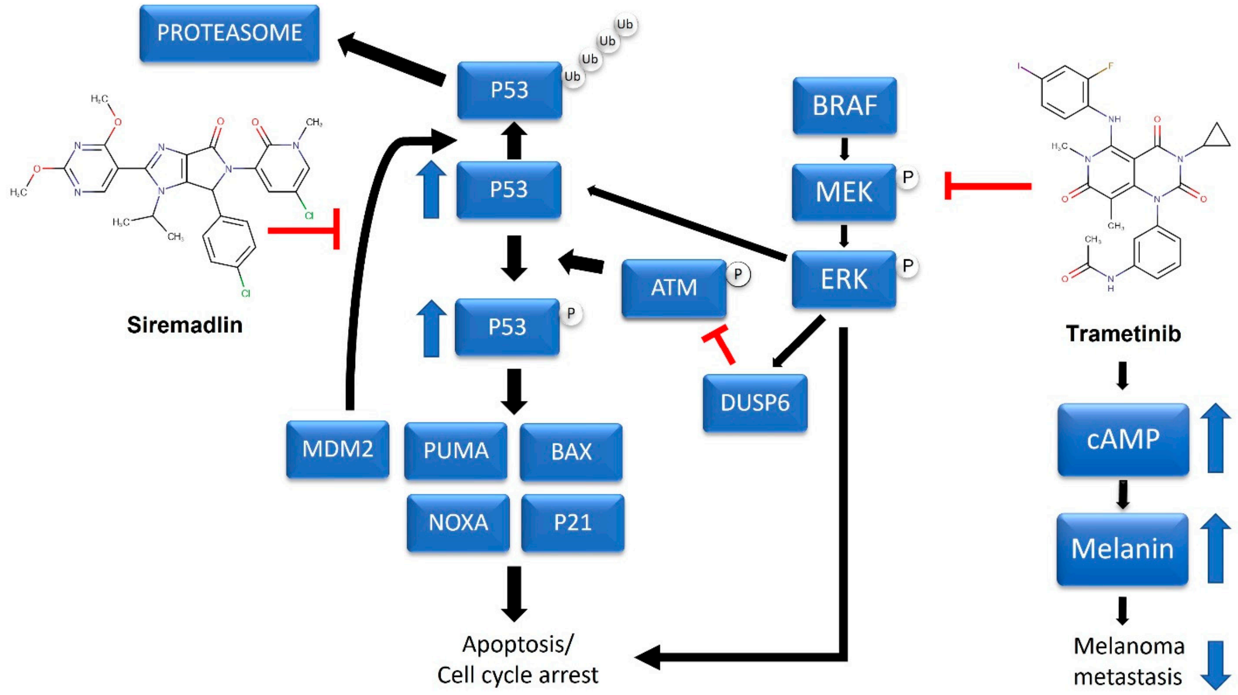

Figure S1. Siremadlin and trametinib mechanism of action in BRAFV600E and p53WT melanoma cells. Trametinib may act in two ways. First, increasing intracellular cAMP leading to an increase in melanin production [16,17] which in melanoma cells may limit further metastasis [18,19]. Second, by inhibition of MEK, through ERK lead to DUSP6 suppression followed by increased p53 phosphorylation mediated by ATM [20]. These changes, leading to increased p53 phosphorylation, promote the induction of p53-dependent transcriptional activity of genes encoding PUMA, NOXA, BAX and p21, which are increasing apoptosis ratio and growth inhibition of melanoma cells. Siremadlin in melanoma cells is inhibiting MDM2 binding and ubiquitylation of p53 and its further degradation in proteasome. This is leading to an increase in p53 levels and the release p53 TAD (Trans-Activation Domain) allowing for its further phosphorylation and induction of p53-dependent transcriptional activity leading to melanoma cells apoptosis or cell cycle arrest.

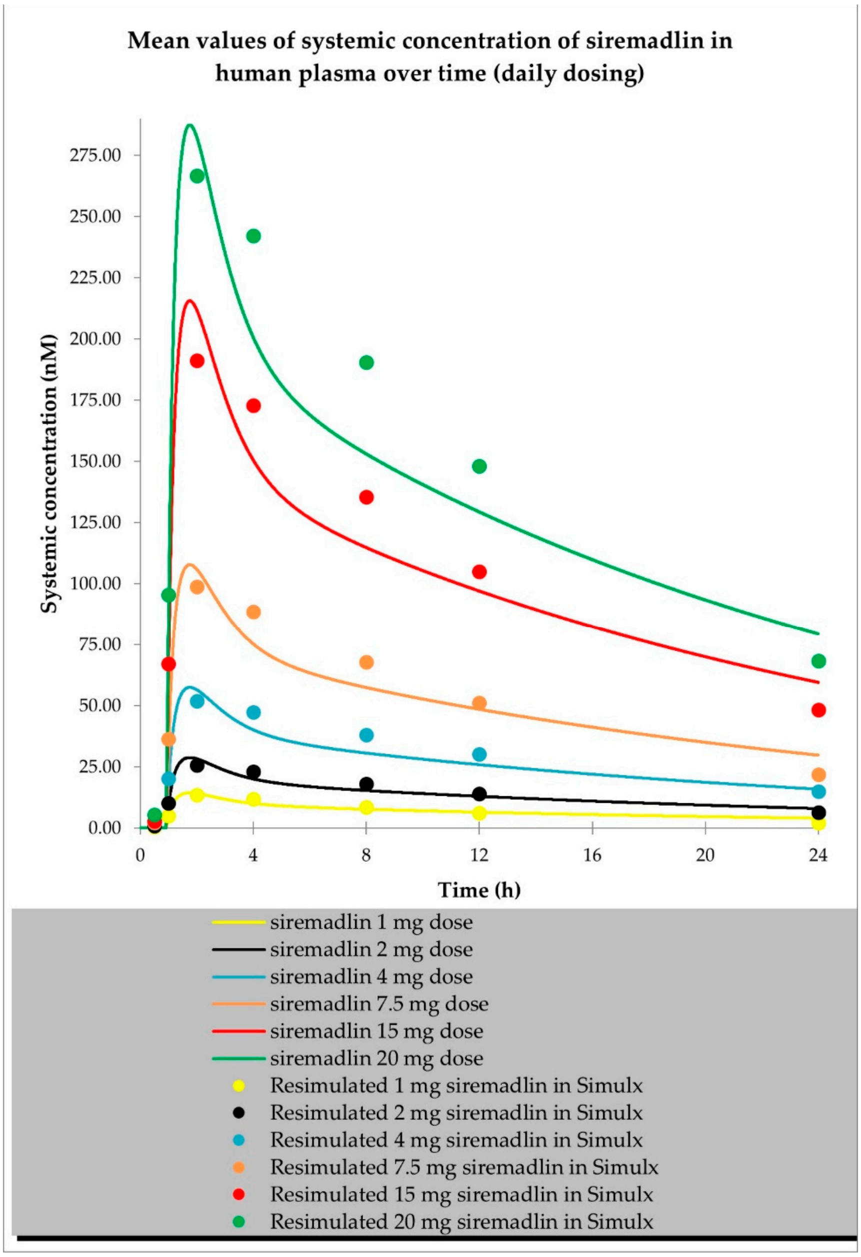

Figure S2. PBPK model of siremadlin administered in a daily regimen in cancer patients representatives using first-order absorption mechanism. Resimulated data is presented as a geometric mean from number of study participants × 10 (see Table 7 in Materials and Methods section).

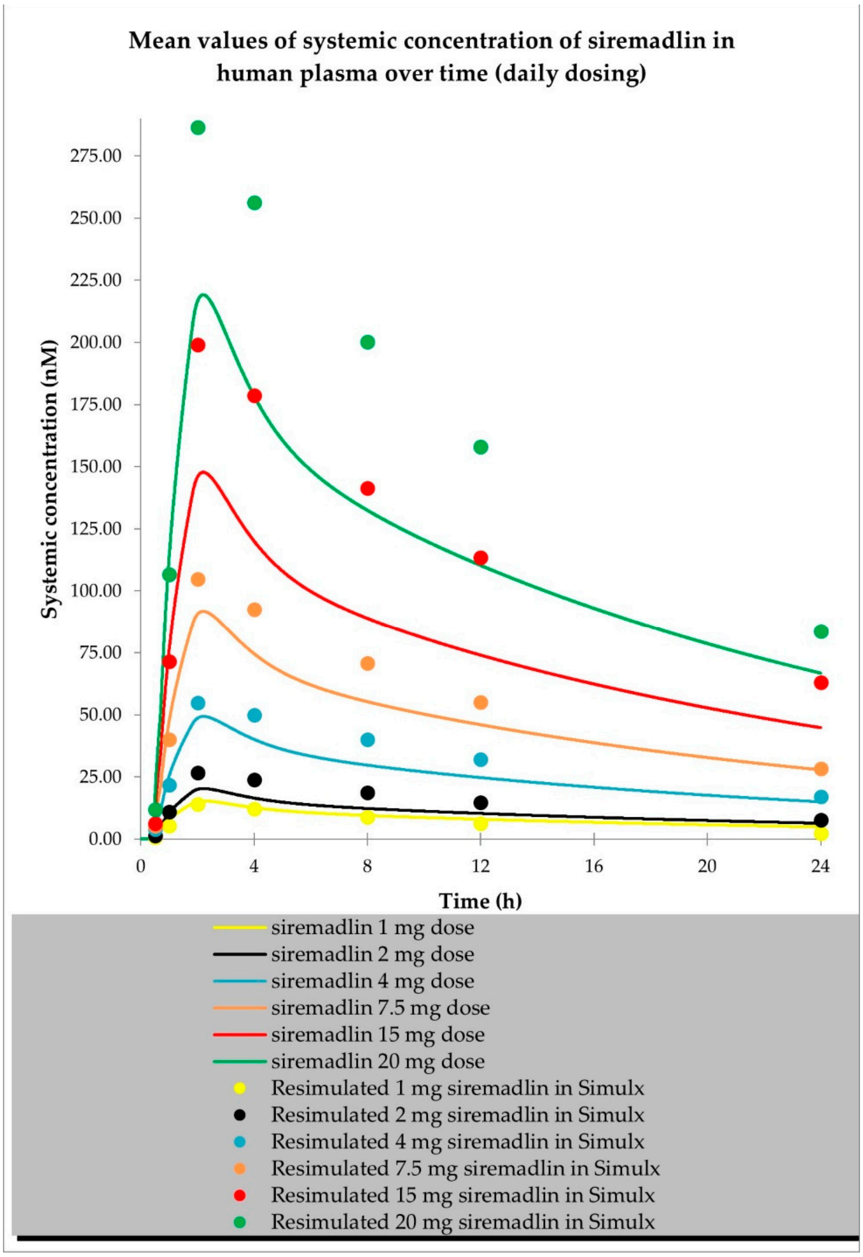

Figure S3. PBPK model of siremadlin administered in daily dosing regimen in cancer patients representatives using mixed zero- and first-order absorption mechanism. Resimulated data is presented as mean from number of study participants × 10 (see Table 7 in Materials and Methods section).

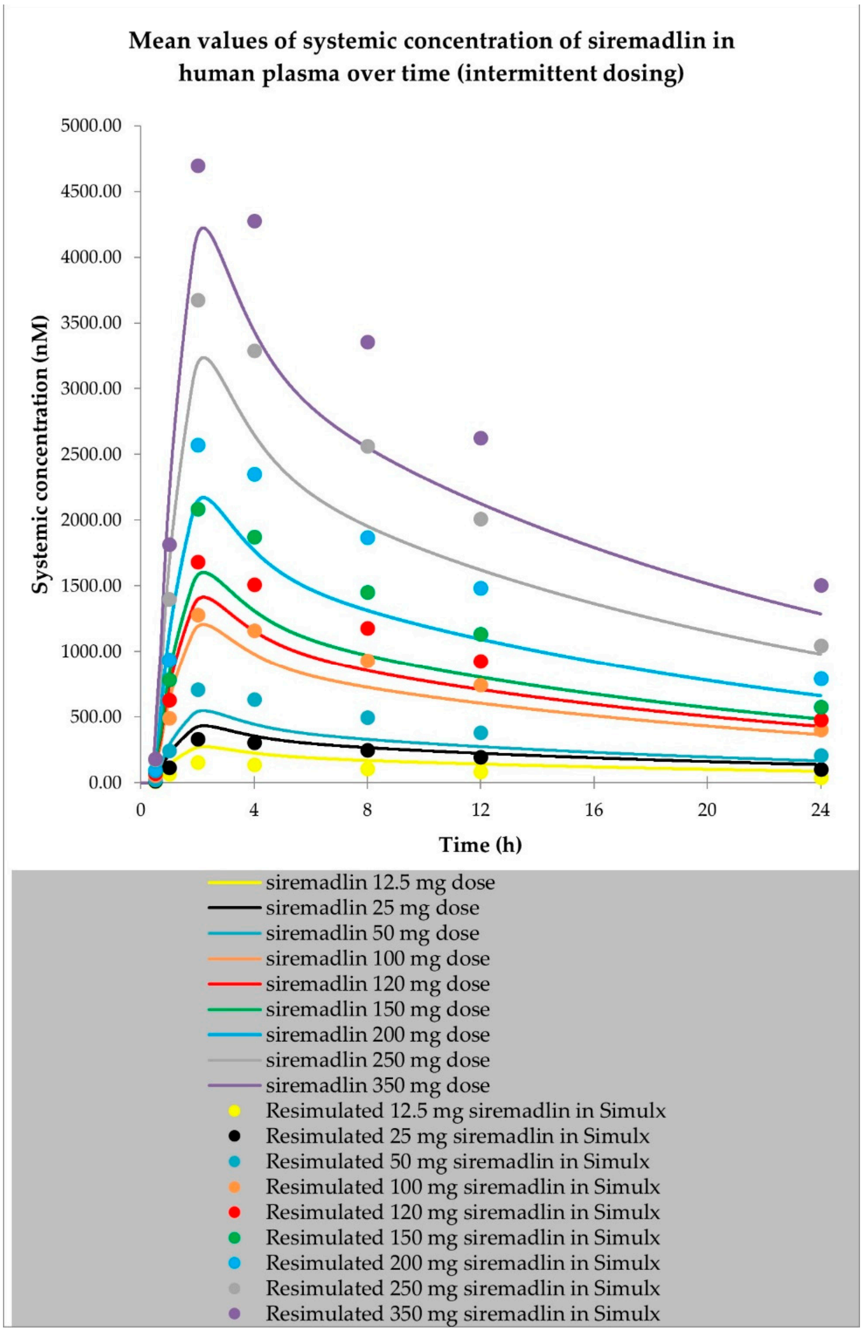

Figure S4. PBPK model of siremadlin administered in intermittent dosing regimen in cancer patients representatives using mixed zero- and first-order absorption mechanism. Resimulated data is presented as mean from number of study participants × 10 (see Table 7 in Materials and Methods section).

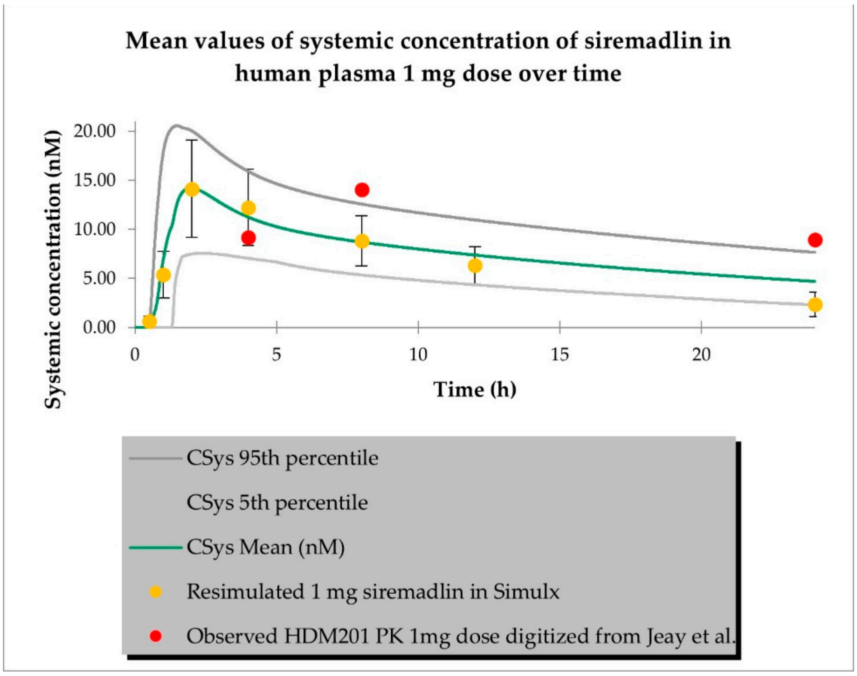

Figure S5. PBPK model of 1 mg dose siremadlin administered in daily regimen in cancer patients population. Resimulated data is presented as mean ± SD from number of study participants (n = 1) n × 10. Observed data was from literature data (data digitized from Jeay et al. [4]).

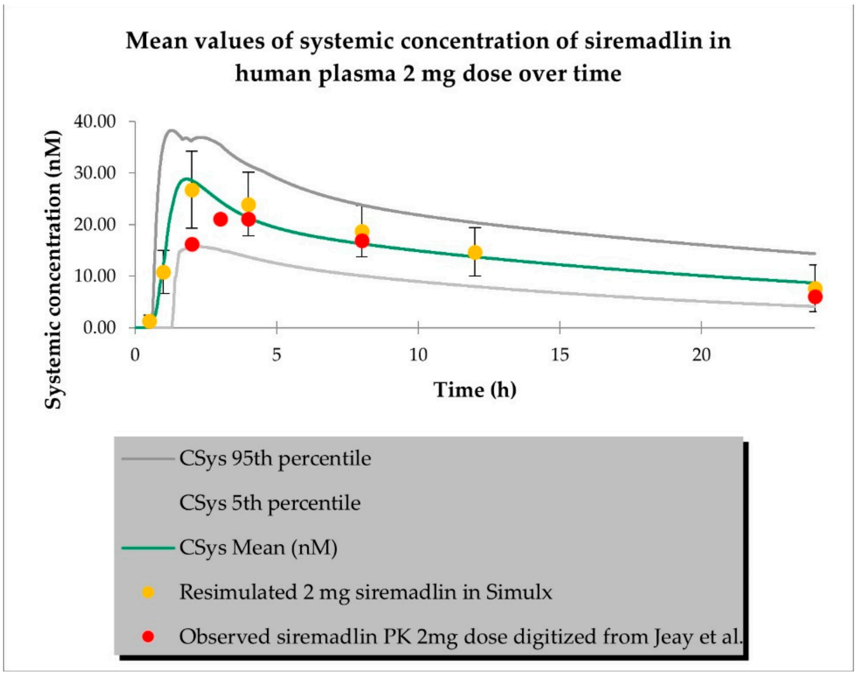

Figure S6. PBPK model of 2 mg dose siremadlin administered in daily regimen in cancer patients population. Resimulated data is presented as mean ± SD from number of study participants (n = 2) n × 10. Observed data was from literature data (data digitized from Jeay et al. [4]).

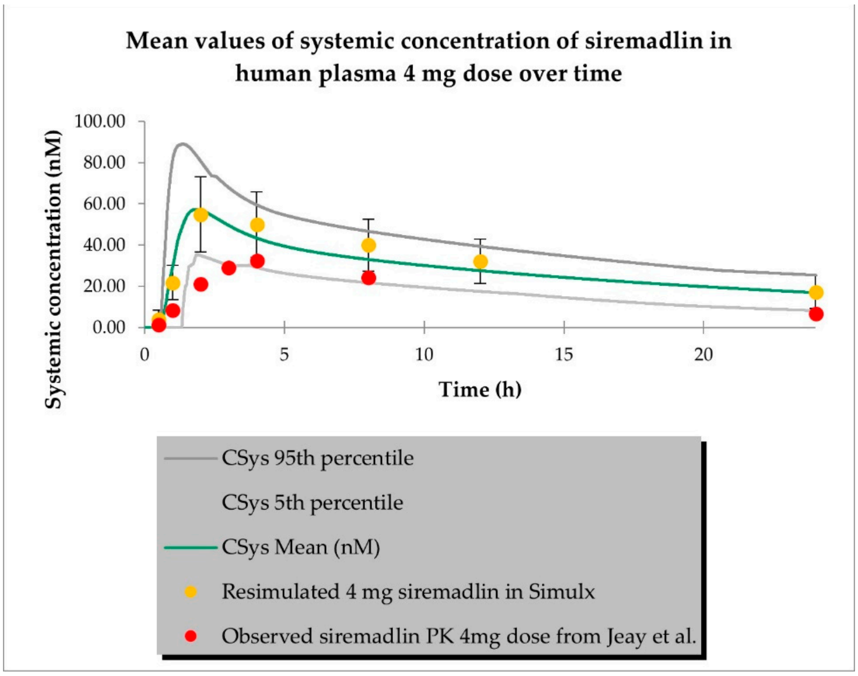

Figure S7. PBPK model of 4 mg dose siremadlin administered in daily regimen in cancer patients population. Resimulated data is presented as mean ± SD from number of study participants (n = 4) n × 10. Observed data was from literature data (data digitized from Jeay et al. [4]).

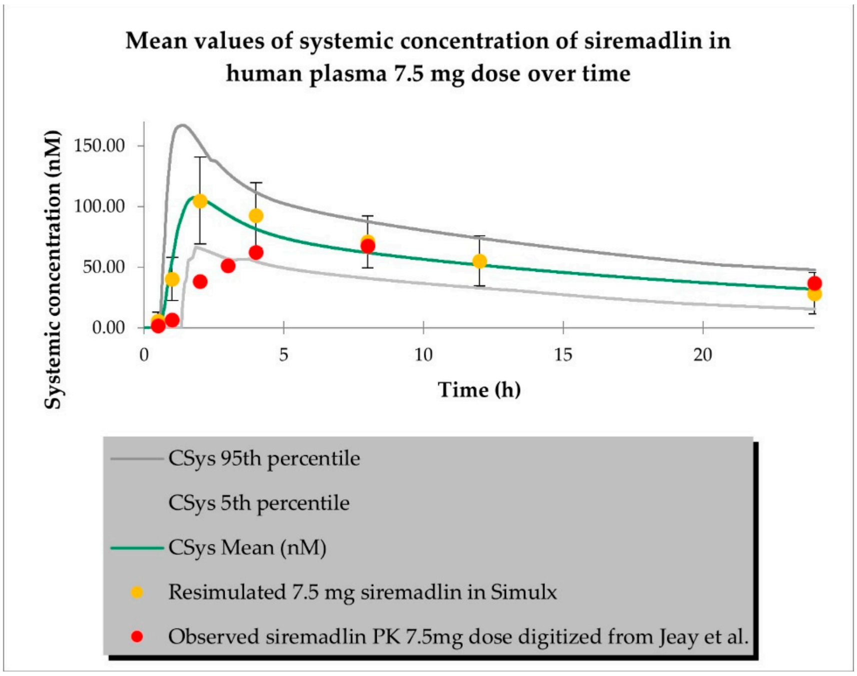

Figure S8. PBPK model of 7.5 mg dose siremadlin administered in daily regimen in cancer patients population. Resimulated data is presented as mean ± SD from number of study participants (n = 4) n × 10. Observed data was from literature data (data digitized from Jeay et al. [4]).

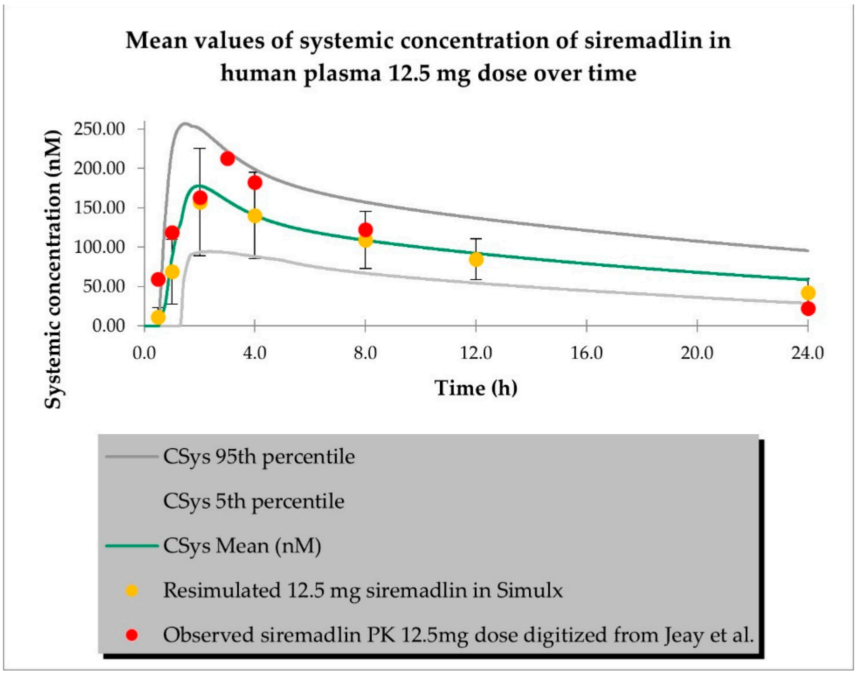

Figure S9. PBPK model of 12.5 mg dose siremadlin administered in intermittent regimen in cancer patients population. Resimulated data is presented as mean ± SD from number of study participants (n = 1) n × 10. Observed data was from literature data (data digitized from Jeay et al. [4]).

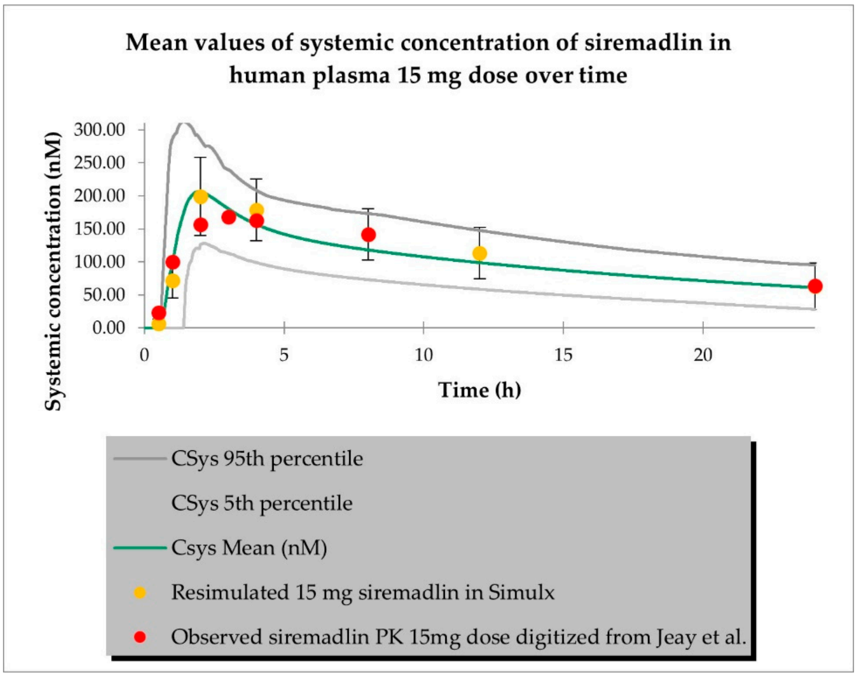

Figure S10. PBPK model of 15 mg dose siremadlin administered in daily regimen in cancer patients population. Resimulated data is presented as mean ± SD from number of study participants (n = 8) n × 10. Observed data was from literature data (data digitized from Jeay et al. [4]).

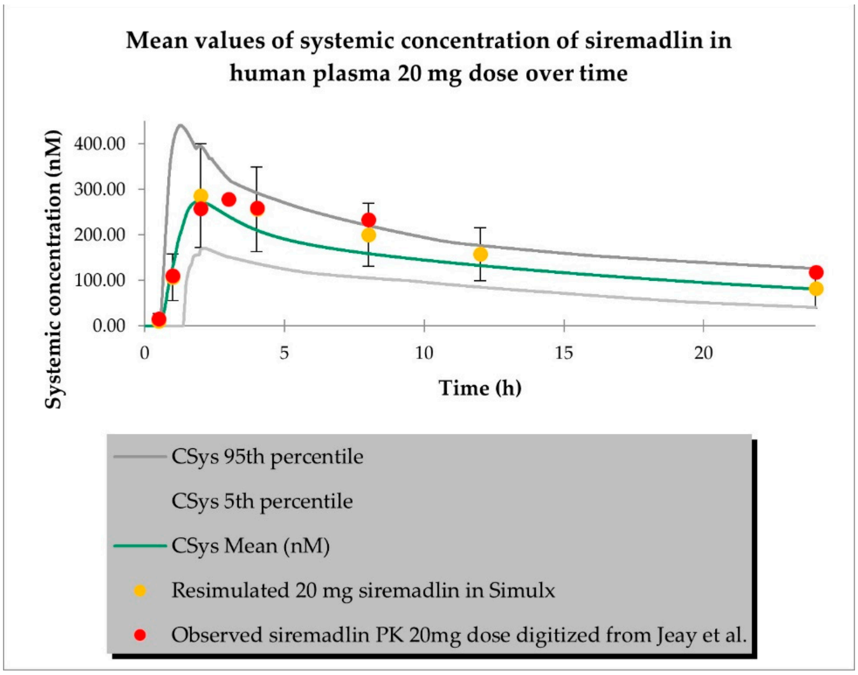

Figure S11. PBPK model of 20 mg dose siremadlin administered in daily regimen in cancer patients population. Resimulated data is presented as mean ± SD from number of study participants (n = 6) n × 10. Observed data was from literature data (data digitized from Jeay et al. [4]).

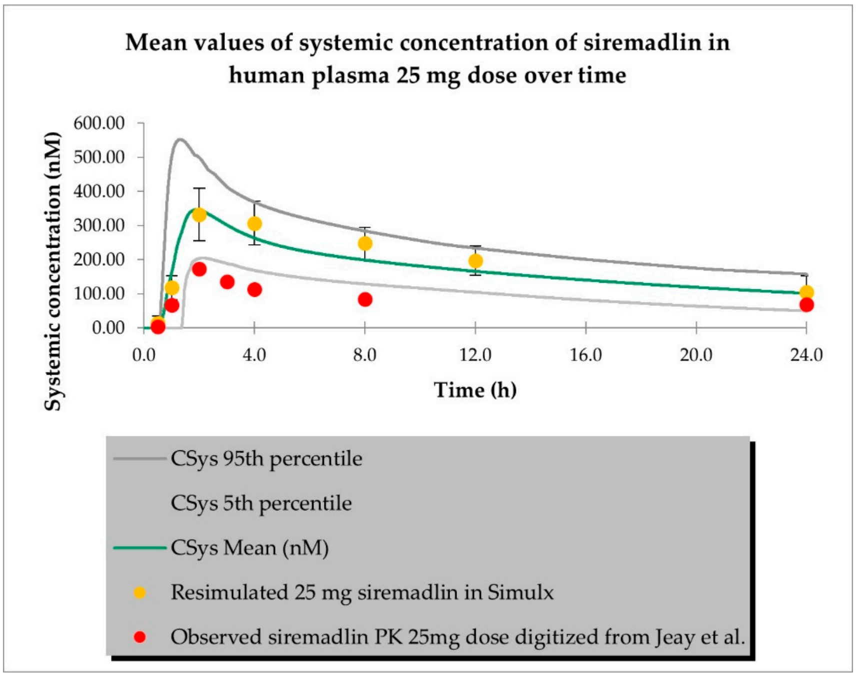

Figure S12. PBPK model of 25 mg dose siremadlin administered in intermittent regimen in cancer patients population. Resimulated data is presented as mean ± SD from number of study participants (n = 5) n × 10. Observed data was from literature data (data digitized from Jeay et al. [4]).

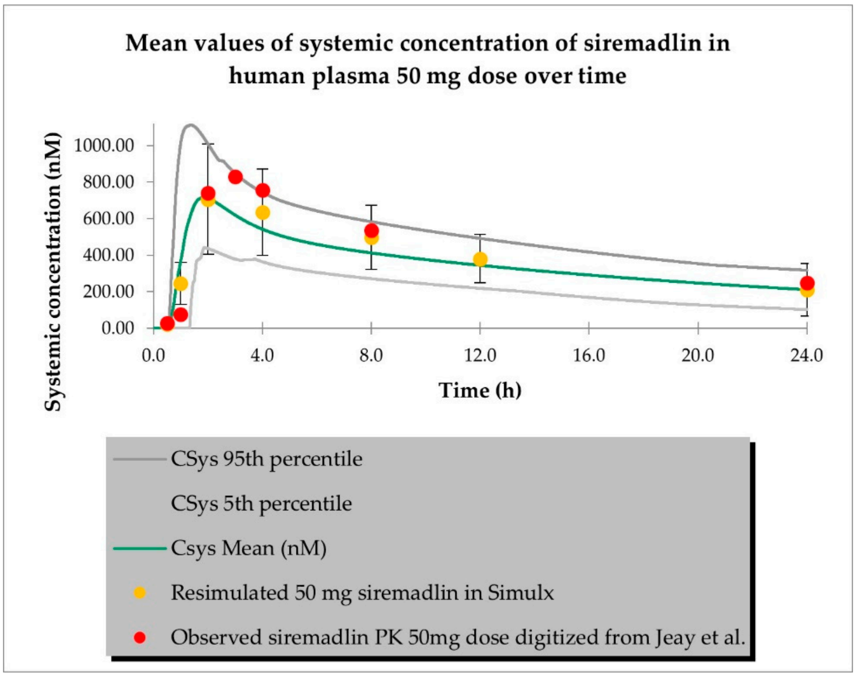

Figure S13. PBPK model of 50 mg dose siremadlin administered in intermittent regimen in cancer patients population. Resimulated data is presented as mean ± SD from number of study participants (n = 4) n × 10. Observed data was from literature data (data digitized from Jeay et al. [4]).

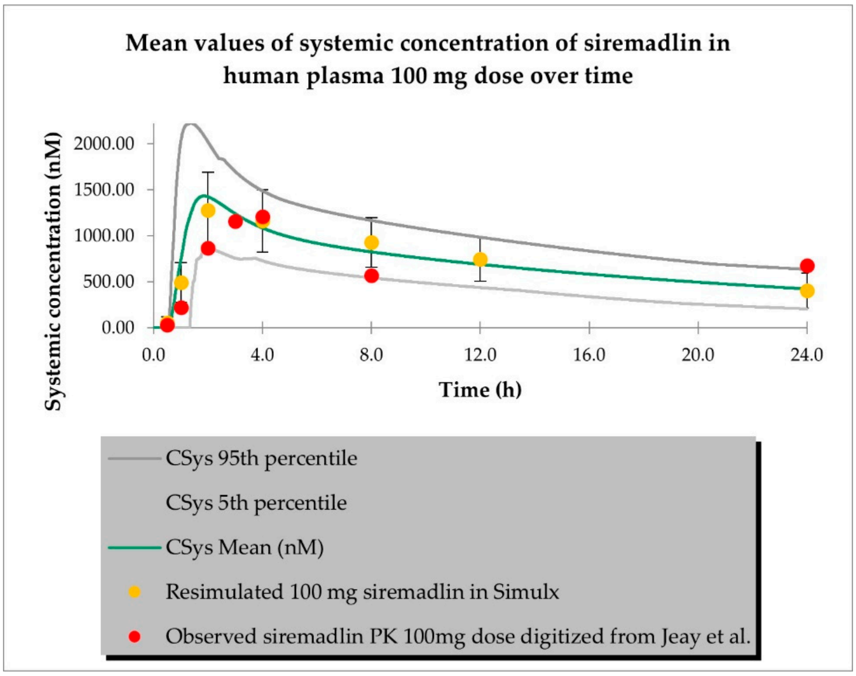

Figure S14. PBPK model of 100 mg dose siremadlin administered in intermittent regimen in cancer patients population. Resimulated data is presented as mean ± SD from number of study participants (n = 4) n × 10. Observed data was from literature data (data digitized from Jeay et al. [4]).

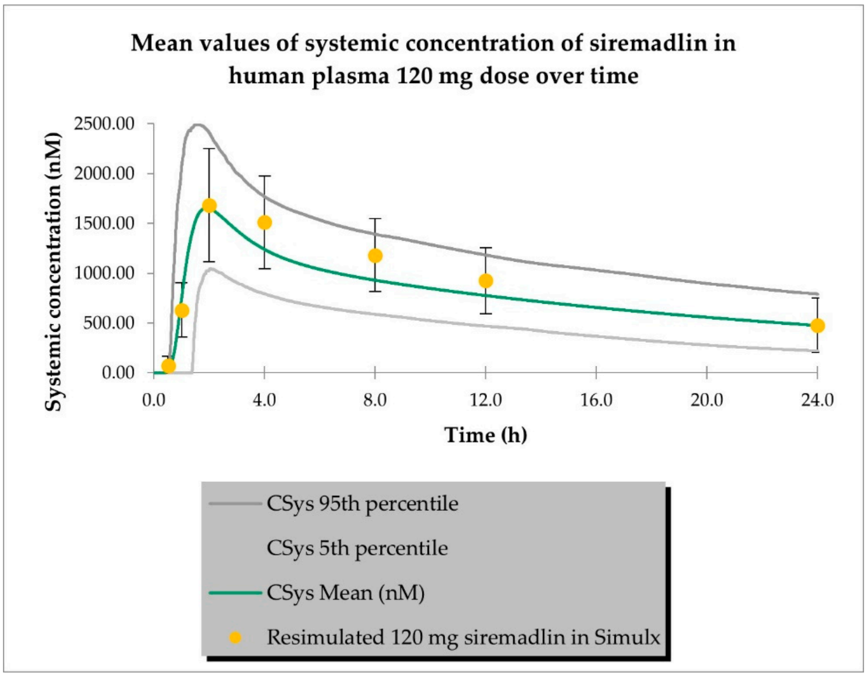

Figure S15. PBPK model of 120 mg dose siremadlin administered in intermittent regimen in cancer patients population. Resimulated data is presented as mean ± SD from number of study participants (n = 29) n × 10.

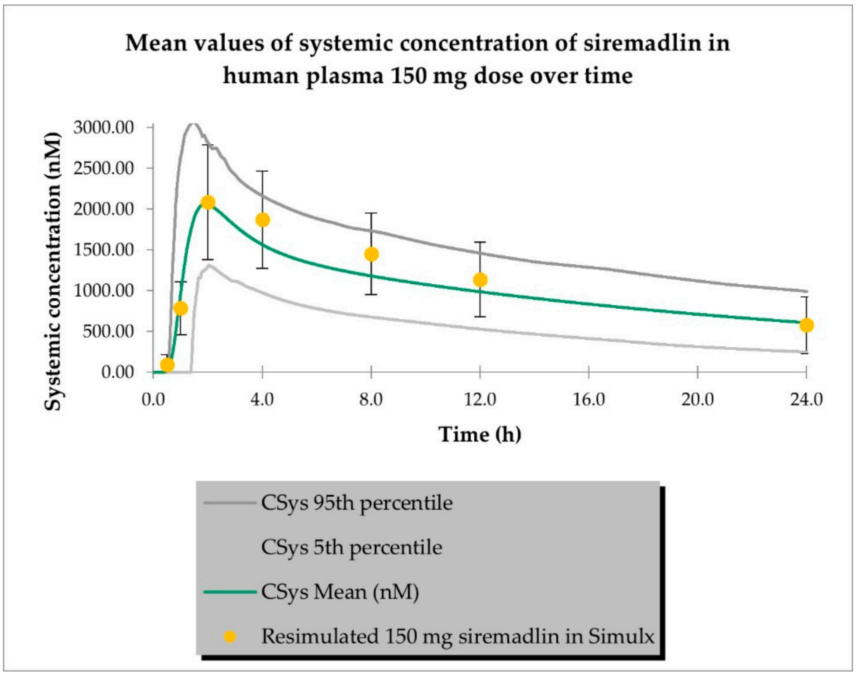

Figure S16. PBPK model of 150 mg dose siremadlin administered in intermittent regimen in cancer patients population. Resimulated data is presented as mean ± SD from number of study participants (n = 15) n × 10.

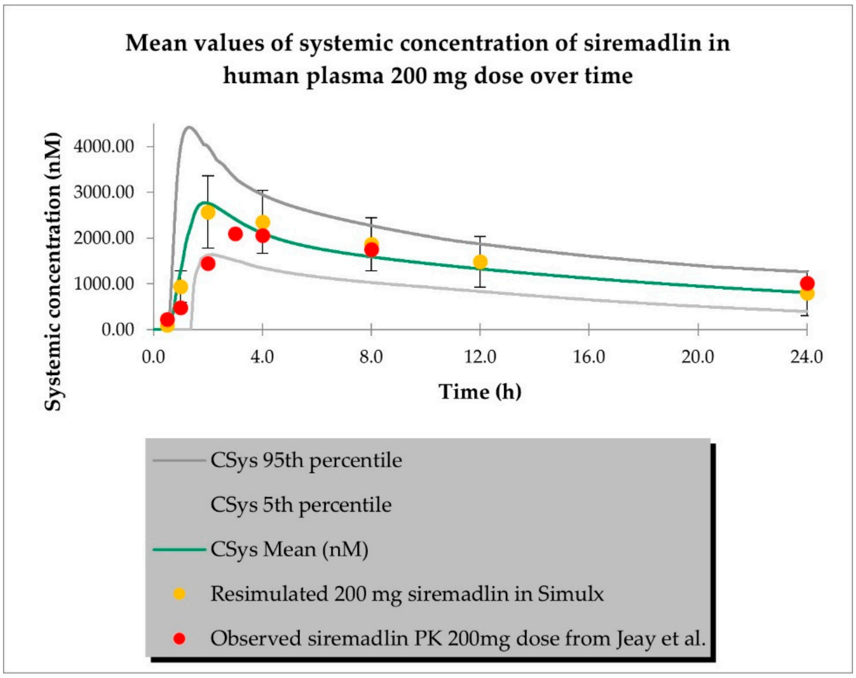

Figure S17. PBPK model of 200 mg dose siremadlin administered in intermittent regimen in cancer patients population. Resimulated data is presented as mean ± SD from number of study participants (n = 5) n × 10. Observed data was from literature data (data digitized from Jeay et al. [4]).

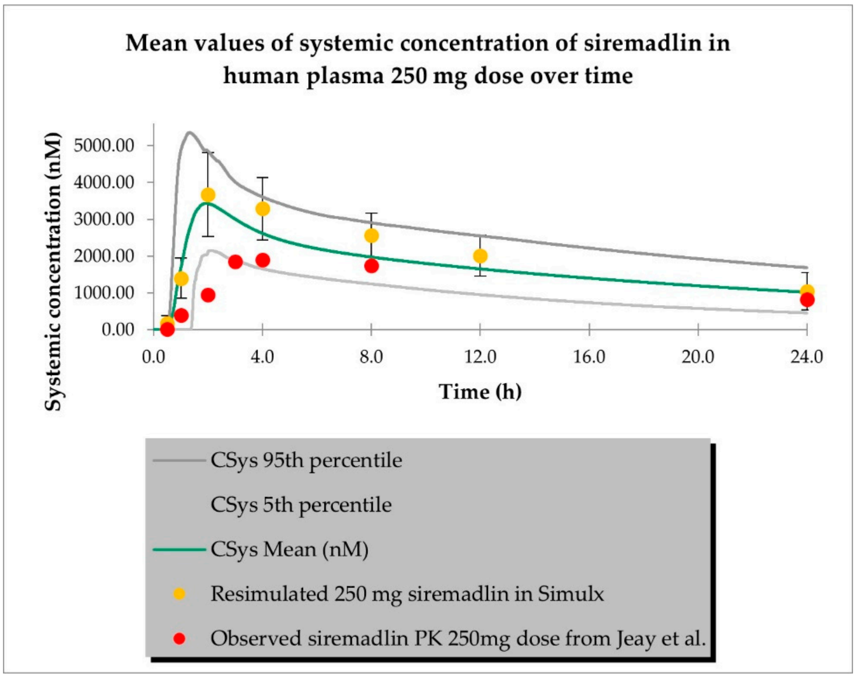

Figure S18. PBPK model of 250 mg dose siremadlin administered in intermittent regimen in cancer patients population. Resimulated data is presented as mean ± SD from number of study participants (n = 9) n × 10. Observed data was from literature data (data digitized from Jeay et al. [4]).

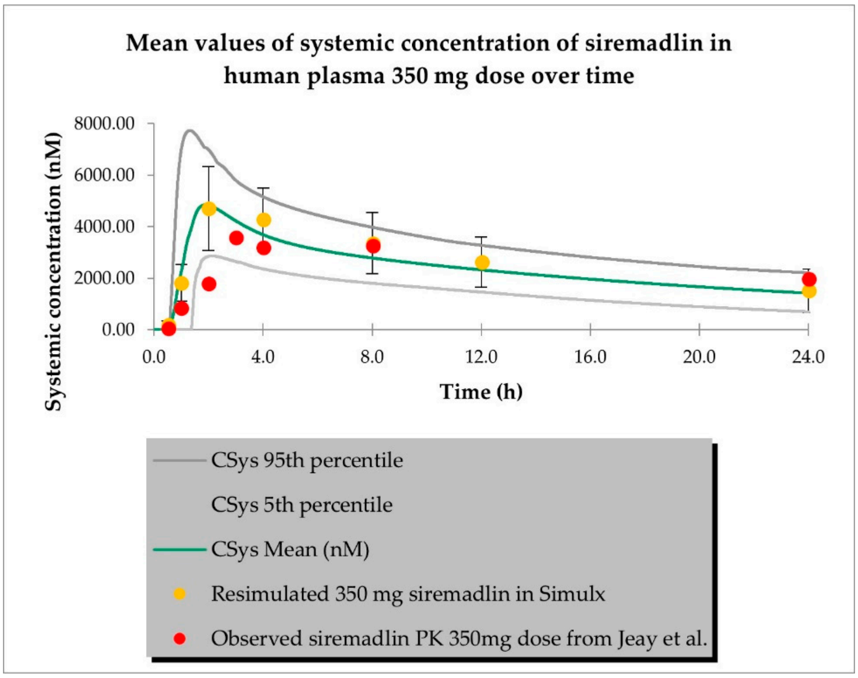

Figure S19. PBPK model of 350 mg dose siremadlin administered in intermittent regimen in cancer patients population. Resimulated data is presented as mean ± SD from number of study participants (n = 5) n × 10. Observed data was from literature data (data digitized from Jeay et al. [4]).

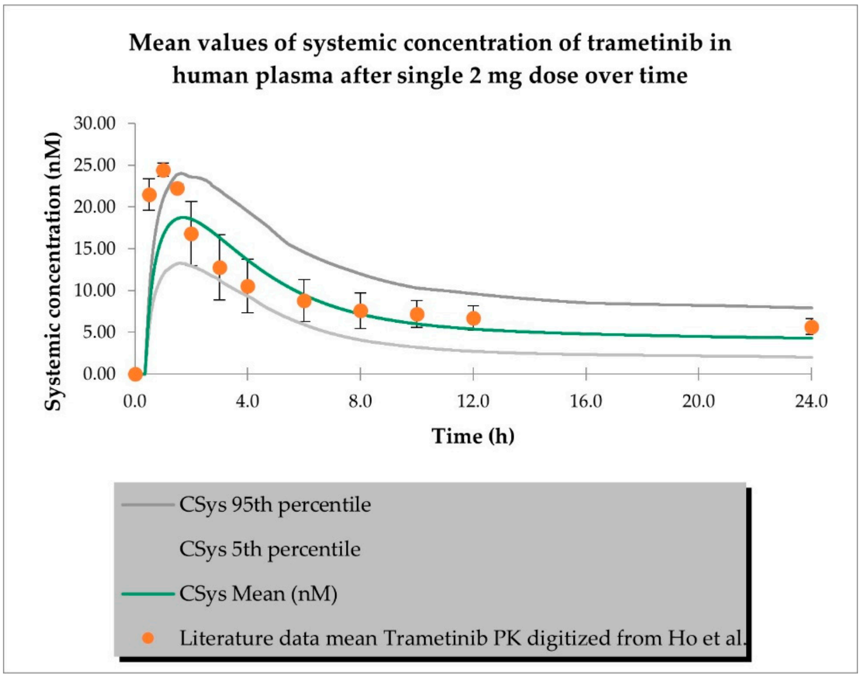

Figure S20. PBPK model of trametinib after single dose in cancer patients population (n = 214). Observed data from literature (data digitized from Infante et al. [21]) is presented as mean ± SD.

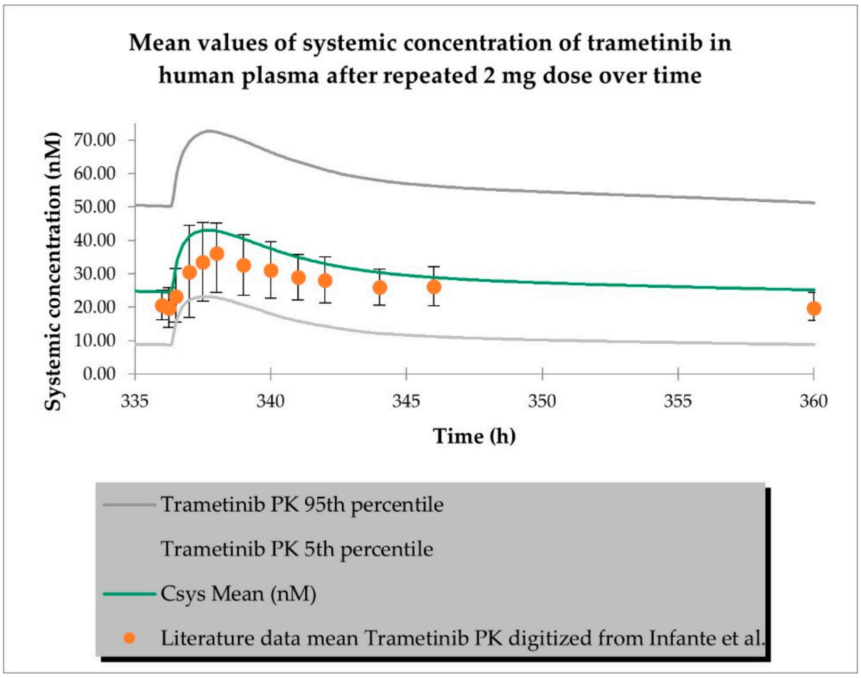

Figure S21. PBPK model of trametinib after repeated dose in cancer patients population (n = 214). Observed data from literature (data digitized from Infante et al. [21]) is presented as mean ± SD.

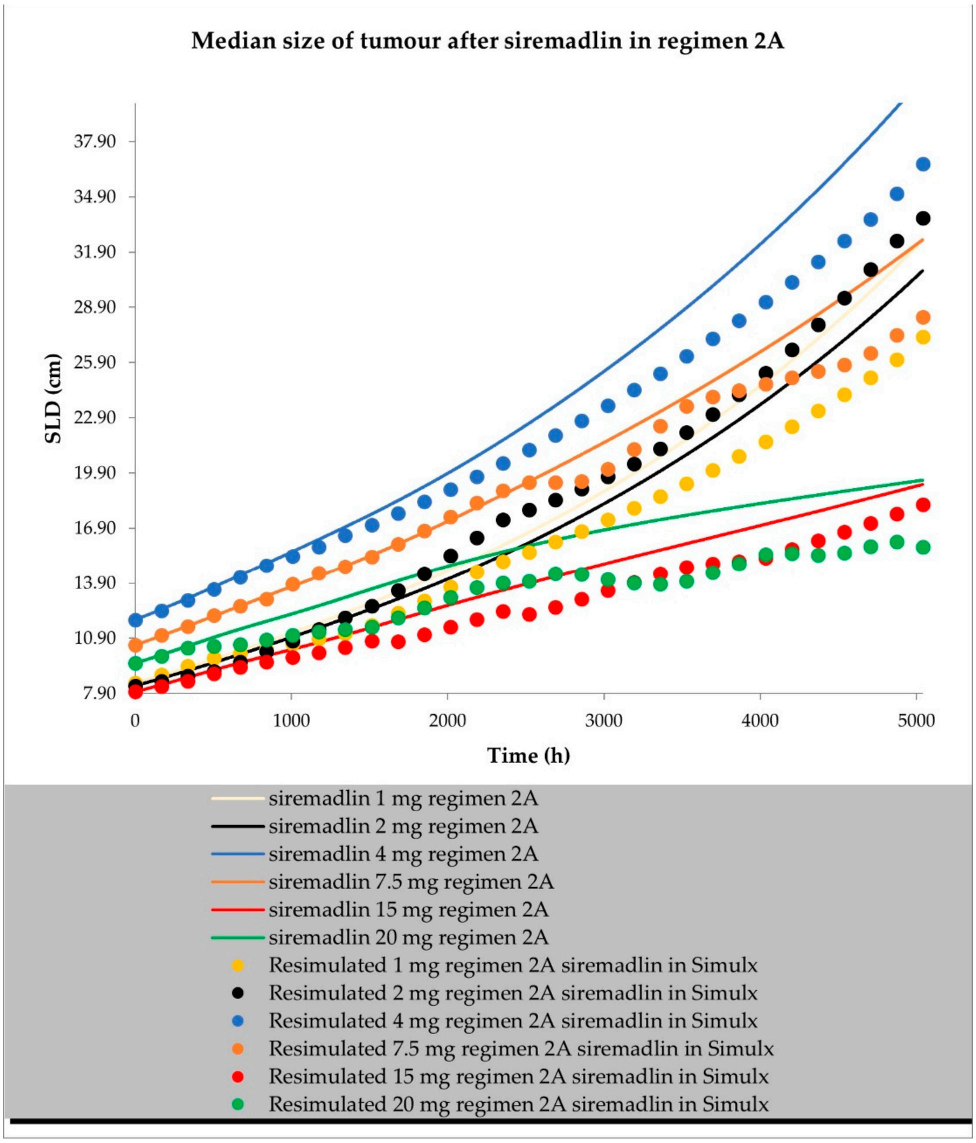

Figure S22. TGI model of siremadlin administered in regimen 2A in cancer patient representatives (n = 1 per treatment arm). Resimulated data is presented as median from number of study participants ×10 (see Table 7).

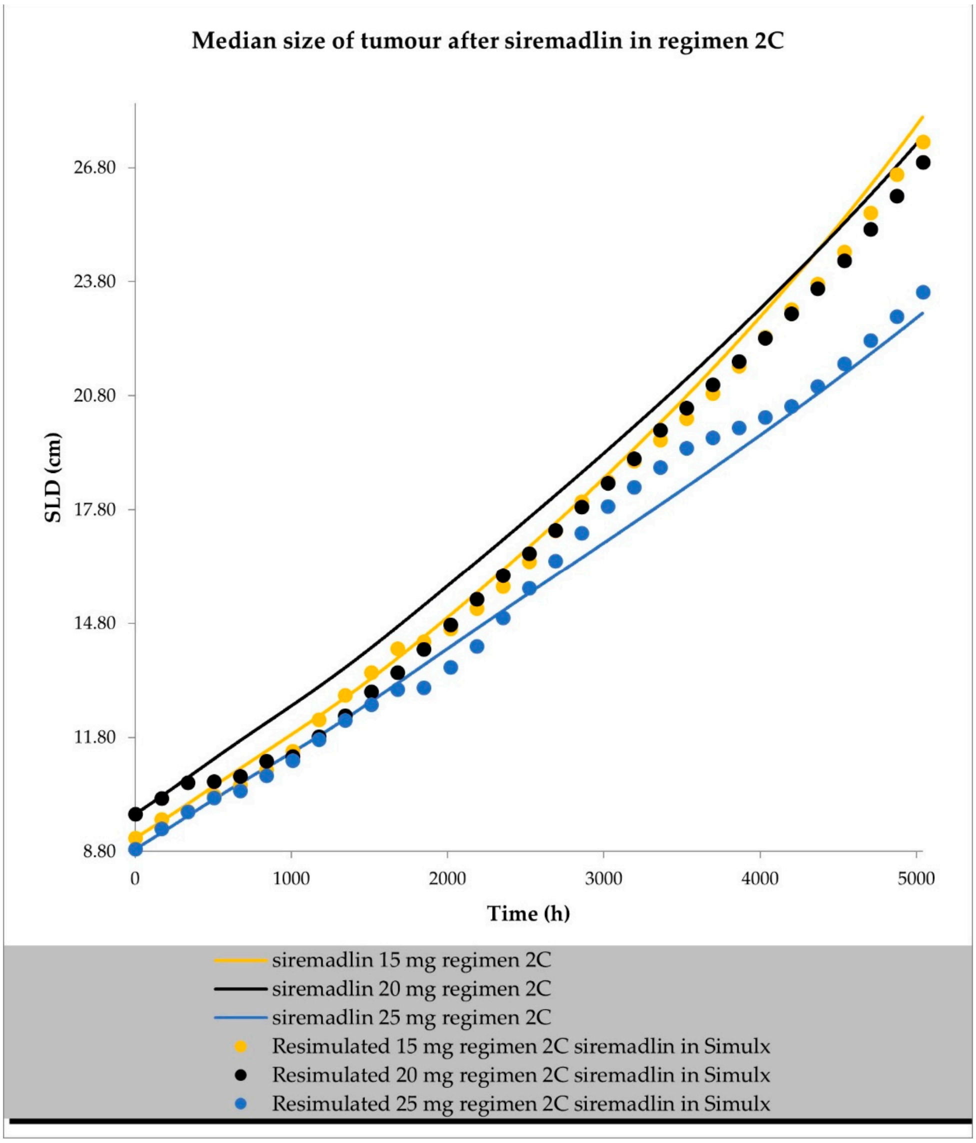

Figure S23. TGI model of siremadlin administered in regimen 2C in cancer patient representatives (n = 1 per treatment arm). Resimulated data is presented as median from number of study participants ×10 (see Table 7).

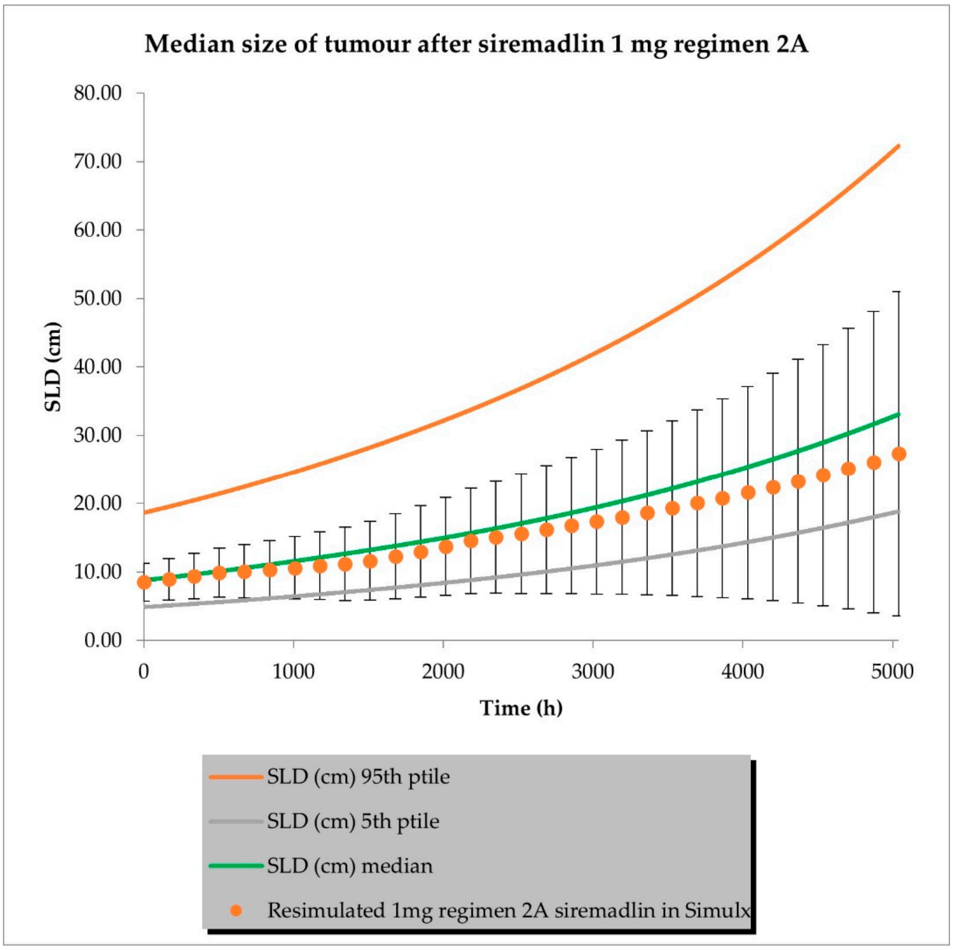

Figure S24. TGI model of 1 mg dose siremadlin administered in daily 2A regimen in cancer patients population. Resimulated data is presented as median ± SE from number of study participants (n = 1) n × 10.

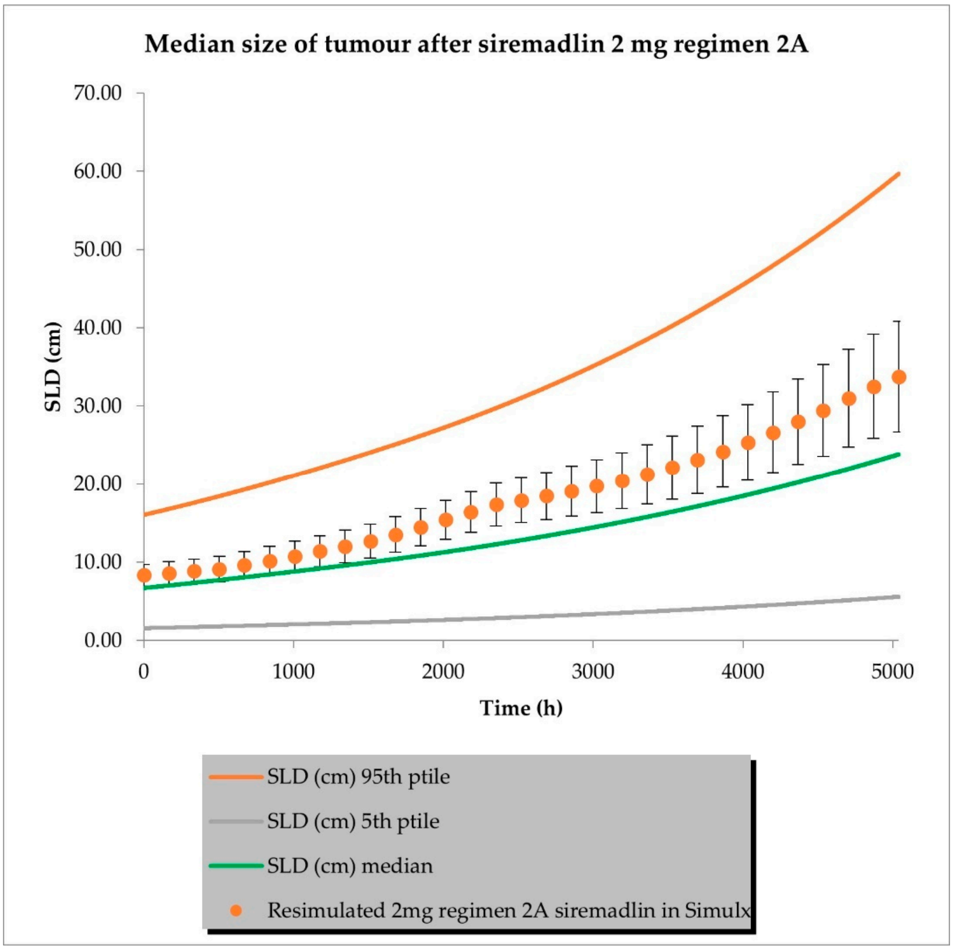

Figure S25. TGI model of 2 mg dose siremadlin administered in daily 2A regimen in cancer patients population. Resimulated data is presented as median ± SE from number of study participants (n = 2) n × 10.

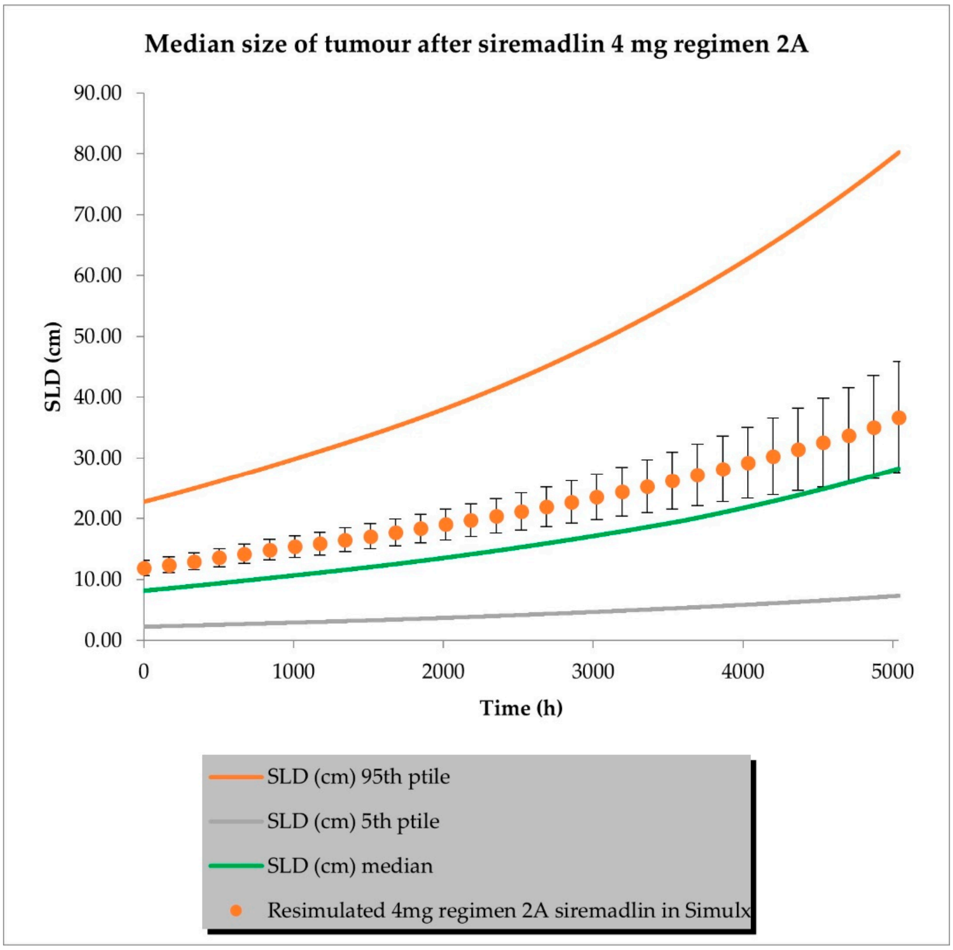

Figure S26. TGI model of 4 mg dose siremadlin administered in daily 2A regimen in cancer patients population. Resimulated data is presented as median ± SE from number of study participants (n = 4) n × 10.

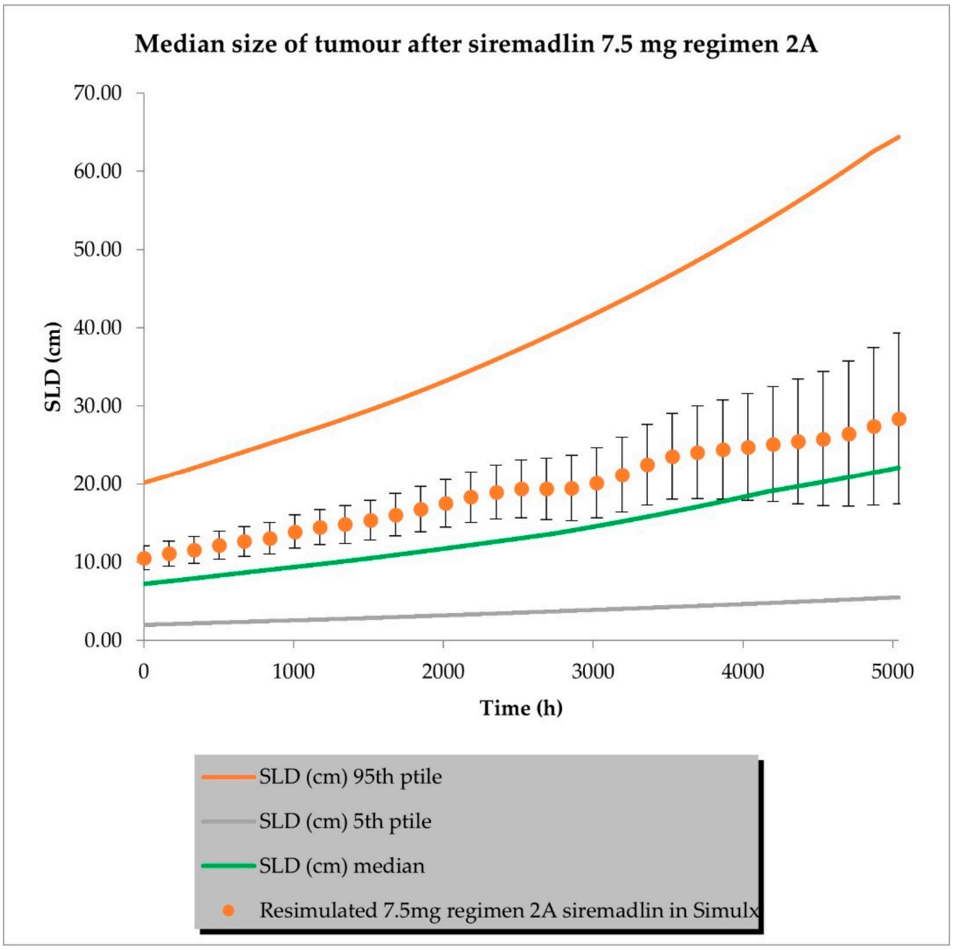

Figure S27. TGI model of 7.5 mg dose siremadlin administered in daily 2A regimen in cancer patients population. Resimulated data is presented as median ± SE from number of study participants (n = 4) n × 10.

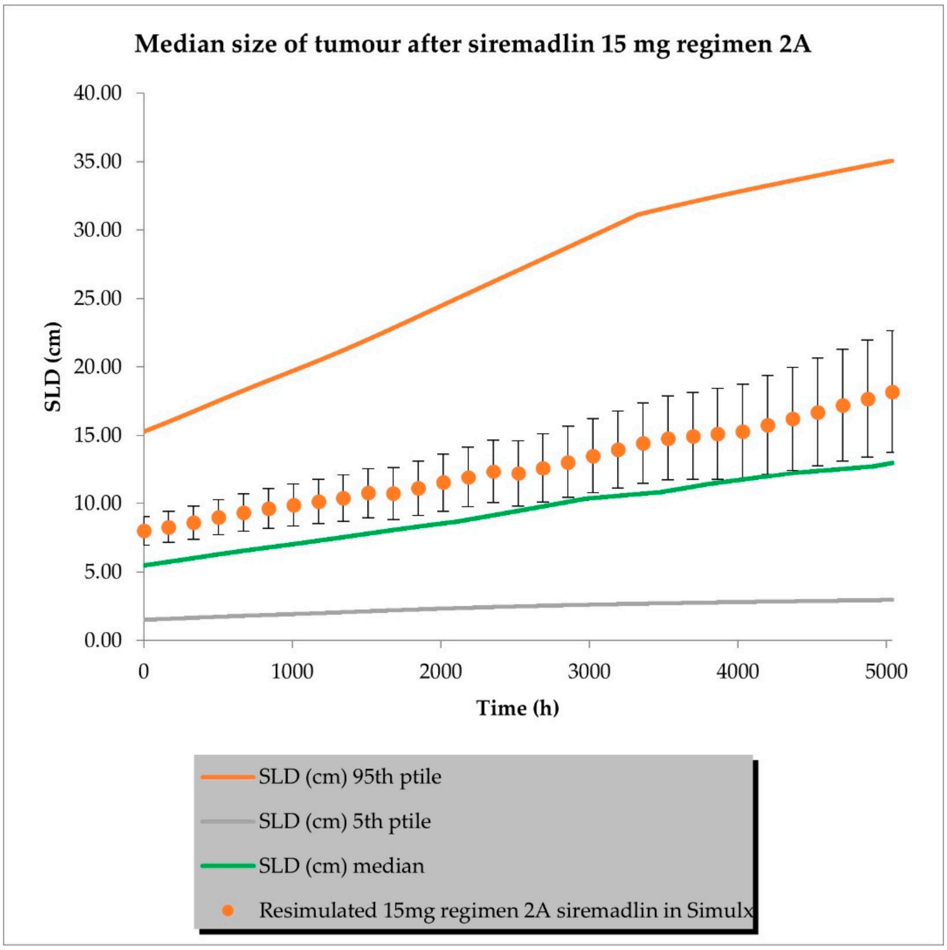

Figure S28. TGI model of 15 mg dose siremadlin administered in daily 2A regimen in cancer patients population. Resimulated data is presented as median ± SE from number of study participants (n = 4) n × 10.

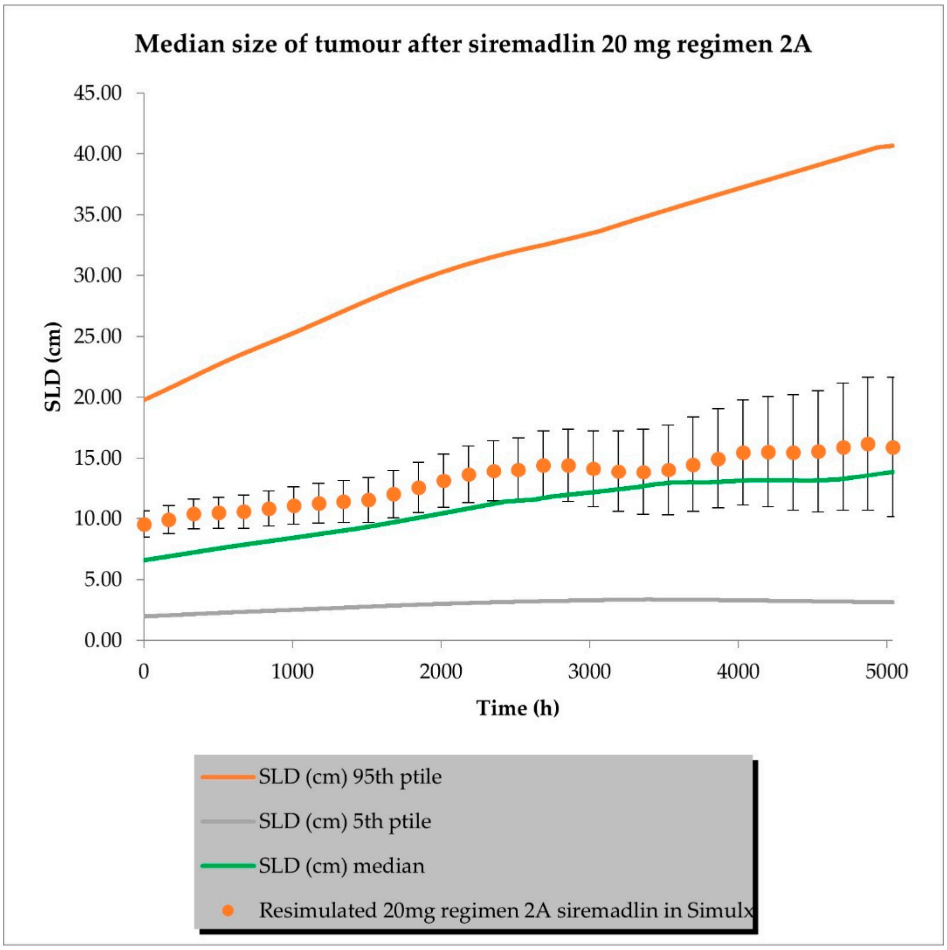

Figure S29. TGI model of 20 mg dose siremadlin administered in daily 2A regimen in cancer patients population. Resimulated data is presented as median ± SE from number of study participants (n = 5) n × 10.

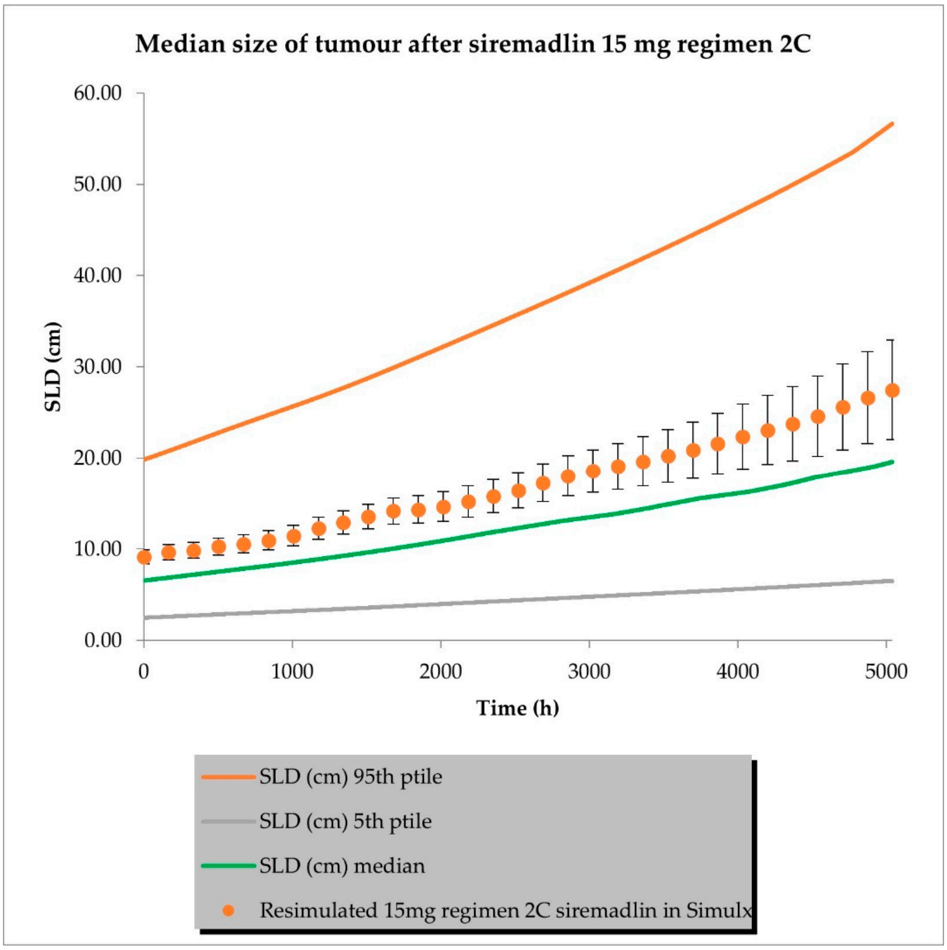

Figure S30. TGI model of 15 mg dose siremadlin administered in daily 2C regimen in cancer patients population. Resimulated data is presented as median ± SE from number of study participants (n = 8) n × 10.

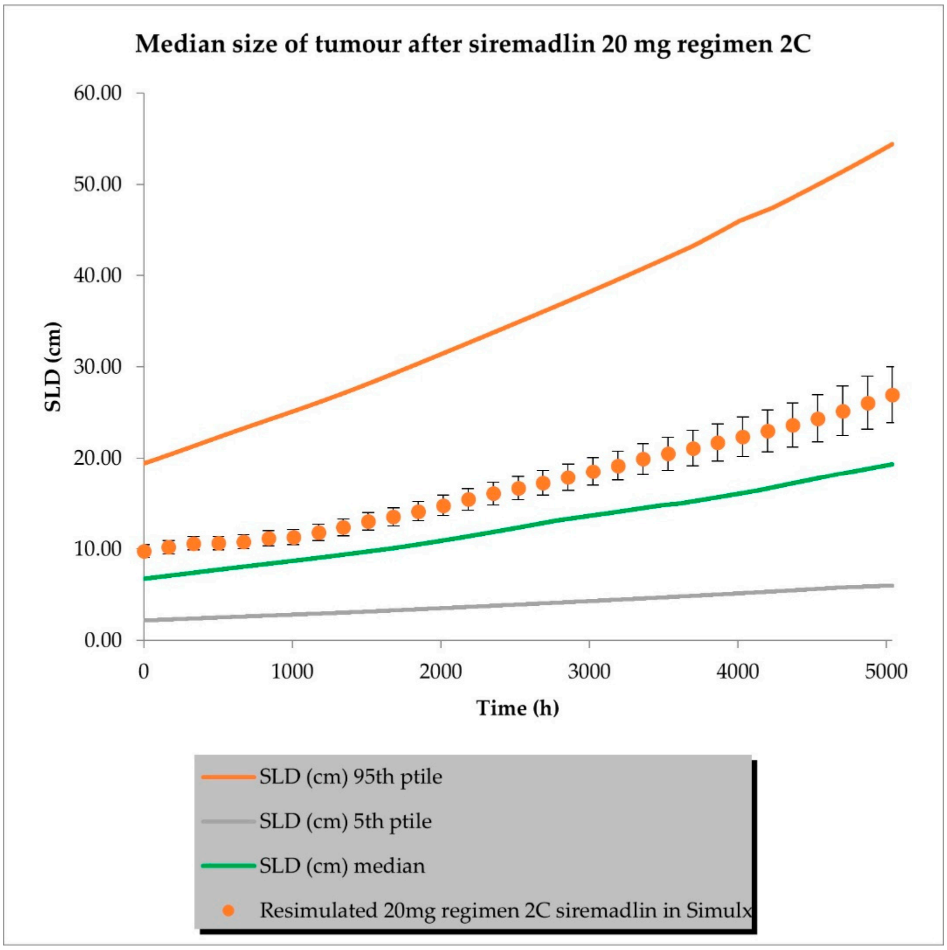

Figure S31. TGI model of 20 mg dose siremadlin administered in daily 2C regimen in cancer patients population. Resimulated data is presented as median ± SE from number of study participants (n = 6) n × 10.

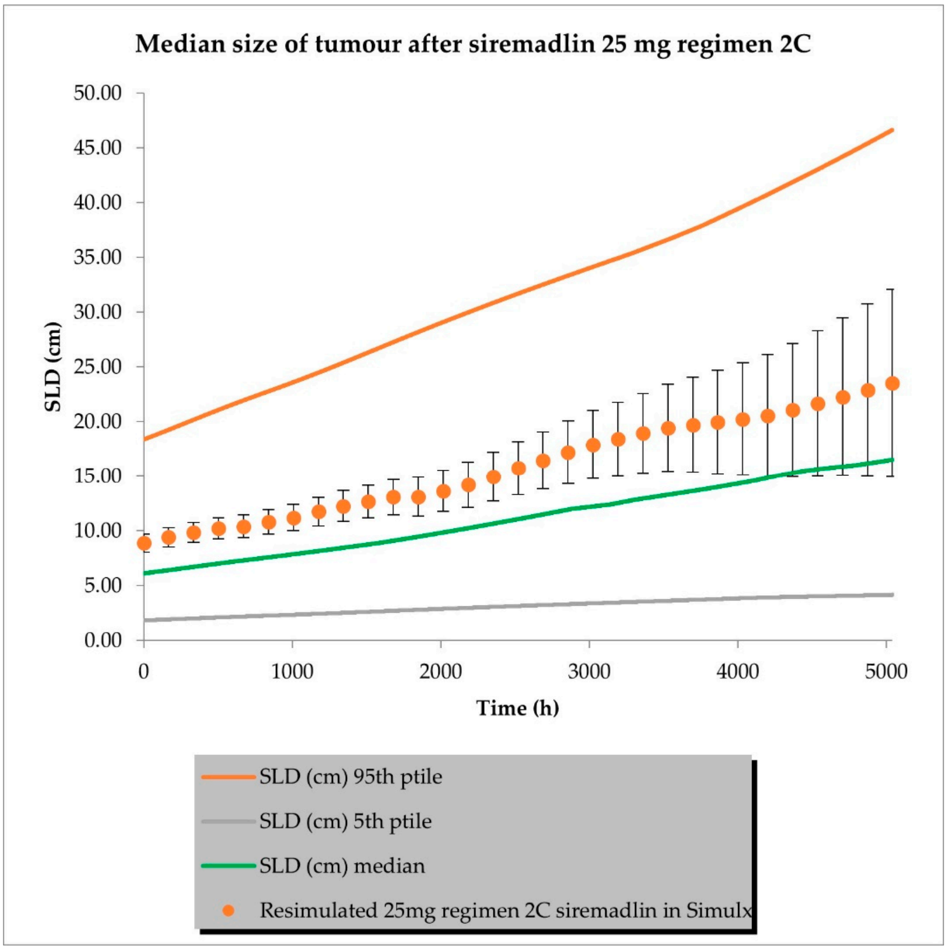

Figure S32. TGI model of 25 mg dose siremadlin administered in daily 2C regimen in cancer patients population. Resimulated data is presented as median ± SE from number of study participants (n = 5) n × 10.

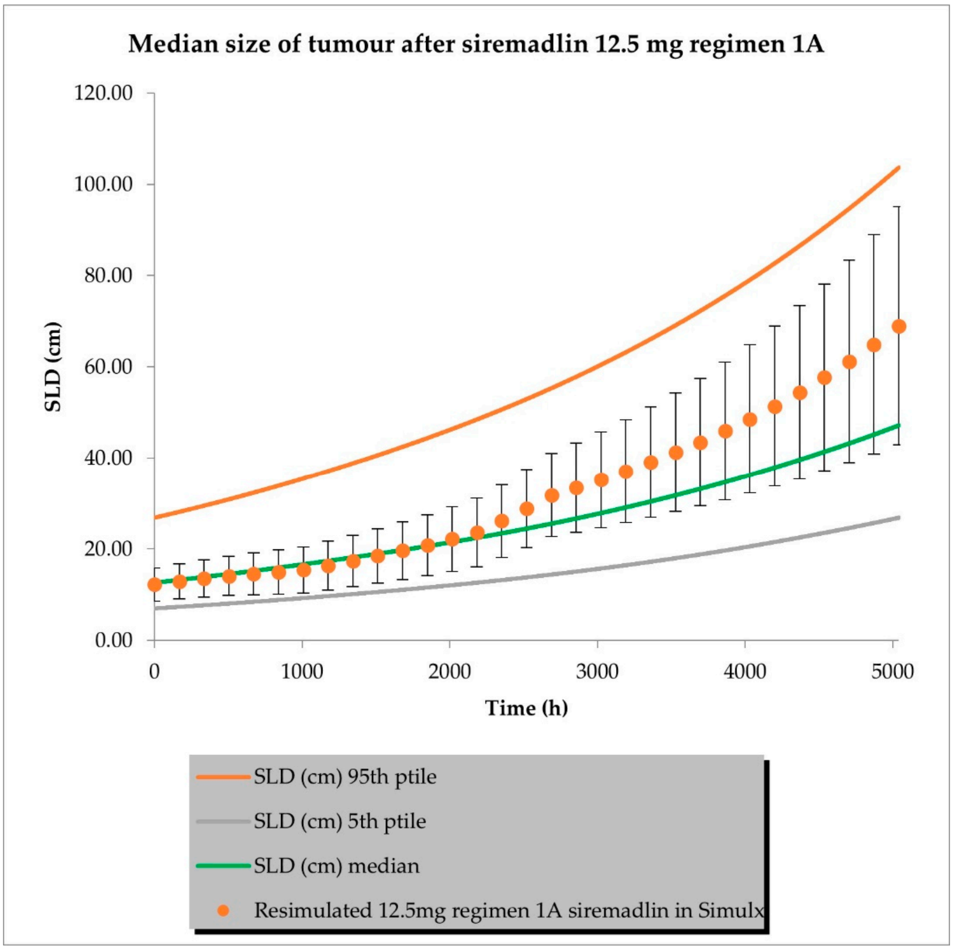

Figure S33. TGI model of 12.5 mg dose siremadlin administered in intermittent 1A regimen in cancer patients population. Resimulated data is presented as median ± SE from number of study participants (n = 1) n × 10.

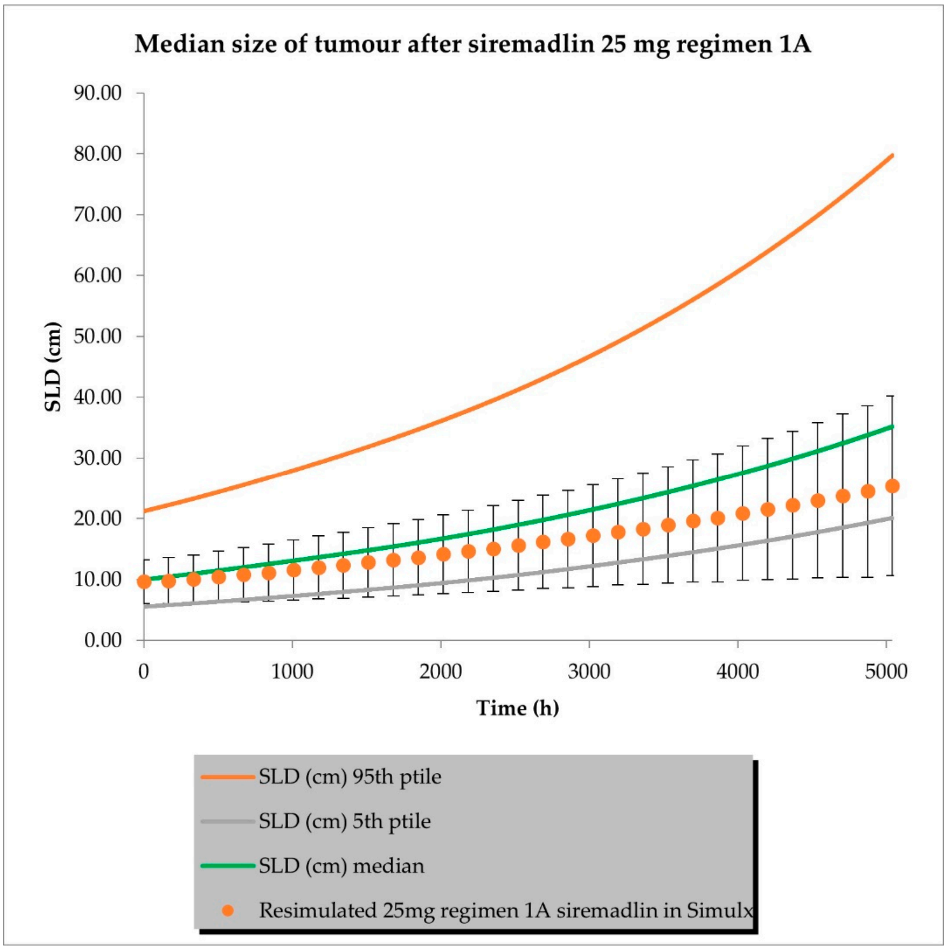

Figure S34. TGI model of 25 mg dose siremadlin administered in intermittent 1A regimen in cancer patients population. Resimulated data is presented as median ± SE from number of study participants (n = 1) n × 10.

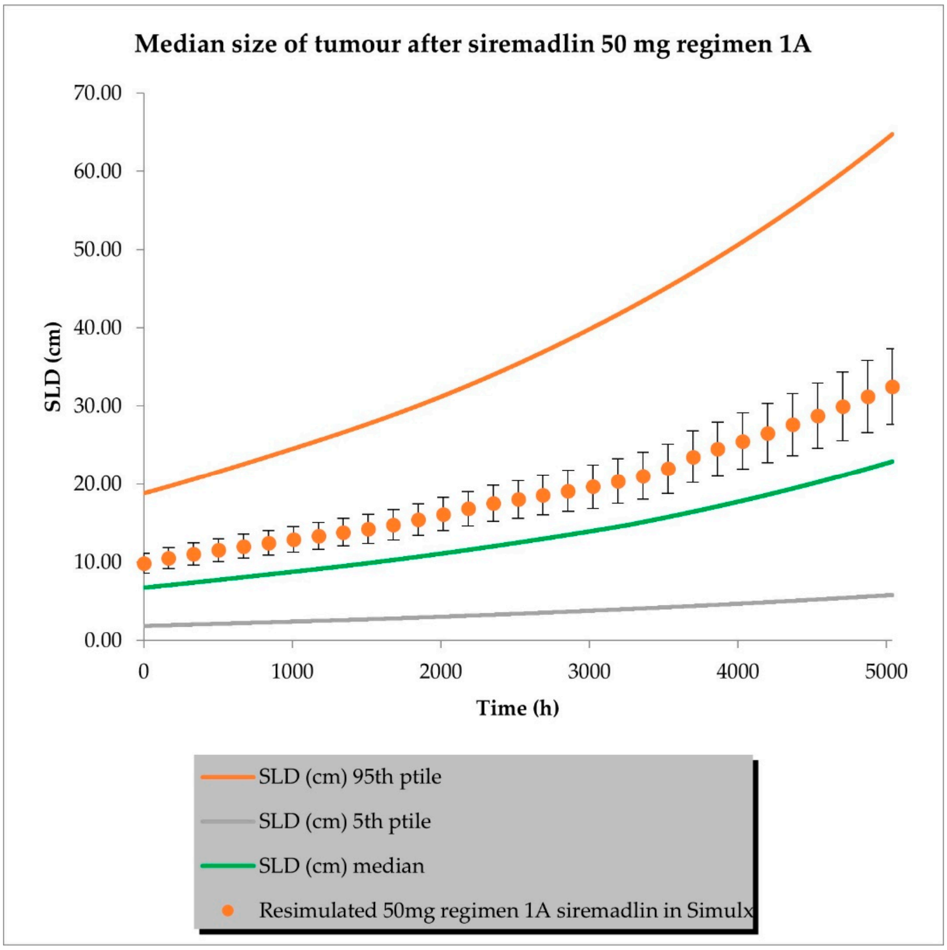

Figure S35. TGI model of 50 mg dose siremadlin administered in intermittent 1A regimen in cancer patients population. Resimulated data is presented as median ± SE from number of study participants (n = 4) n × 10.

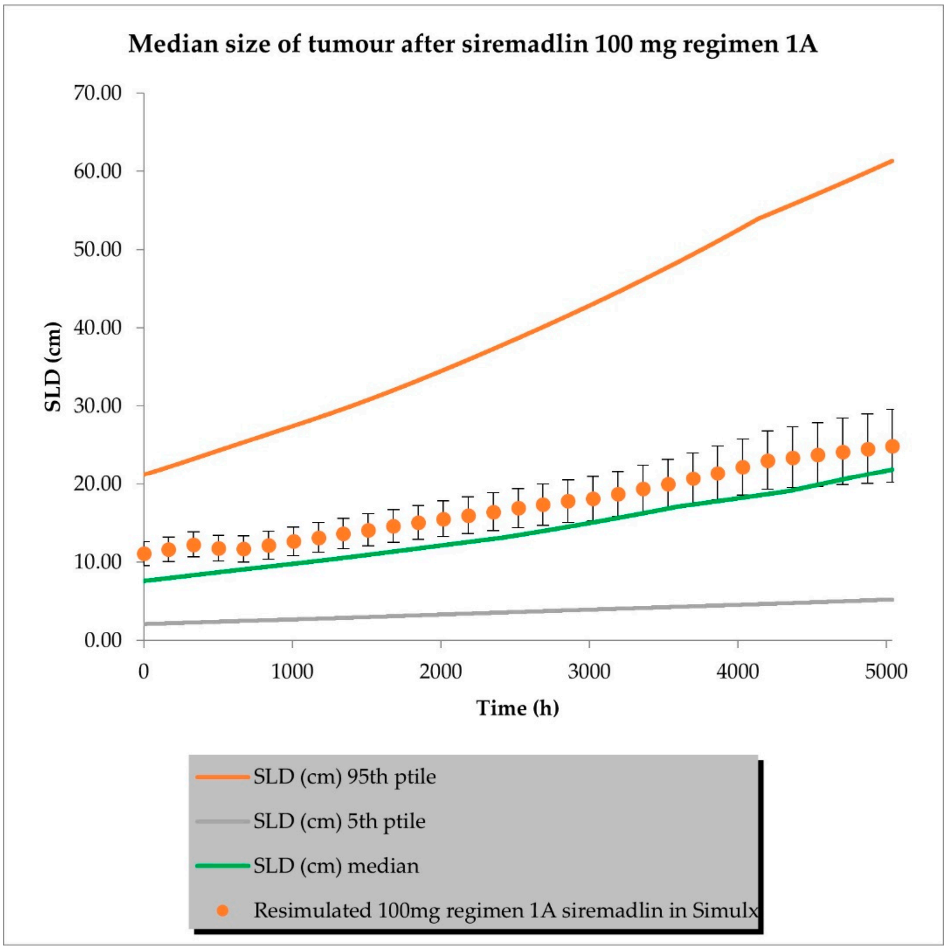

Figure S36. TGI model of 100 mg dose siremadlin administered in intermittent 1A regimen in cancer patients population. Resimulated data is presented as median ± SE from number of study participants (n = 4) n × 10.

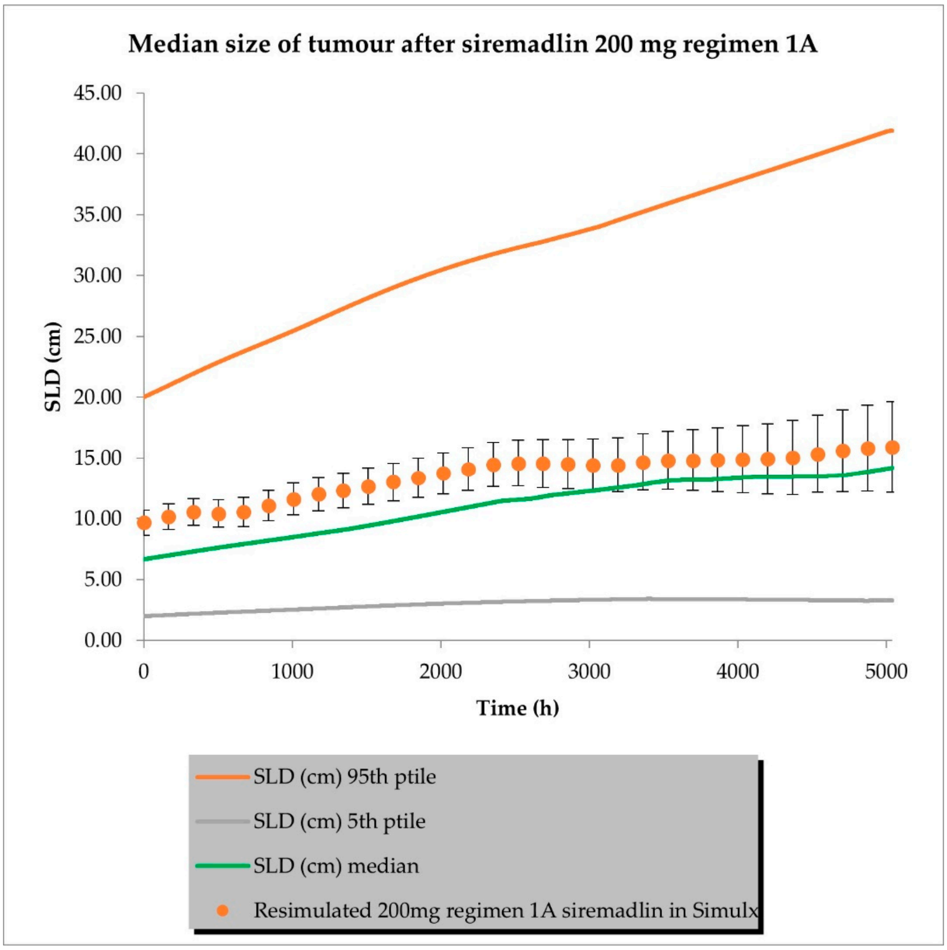

Figure S37. TGI model of 200 mg dose siremadlin administered in intermittent 1A regimen in cancer patients population. Resimulated data is presented as median ± SE from number of study participants (n = 5) n × 10.

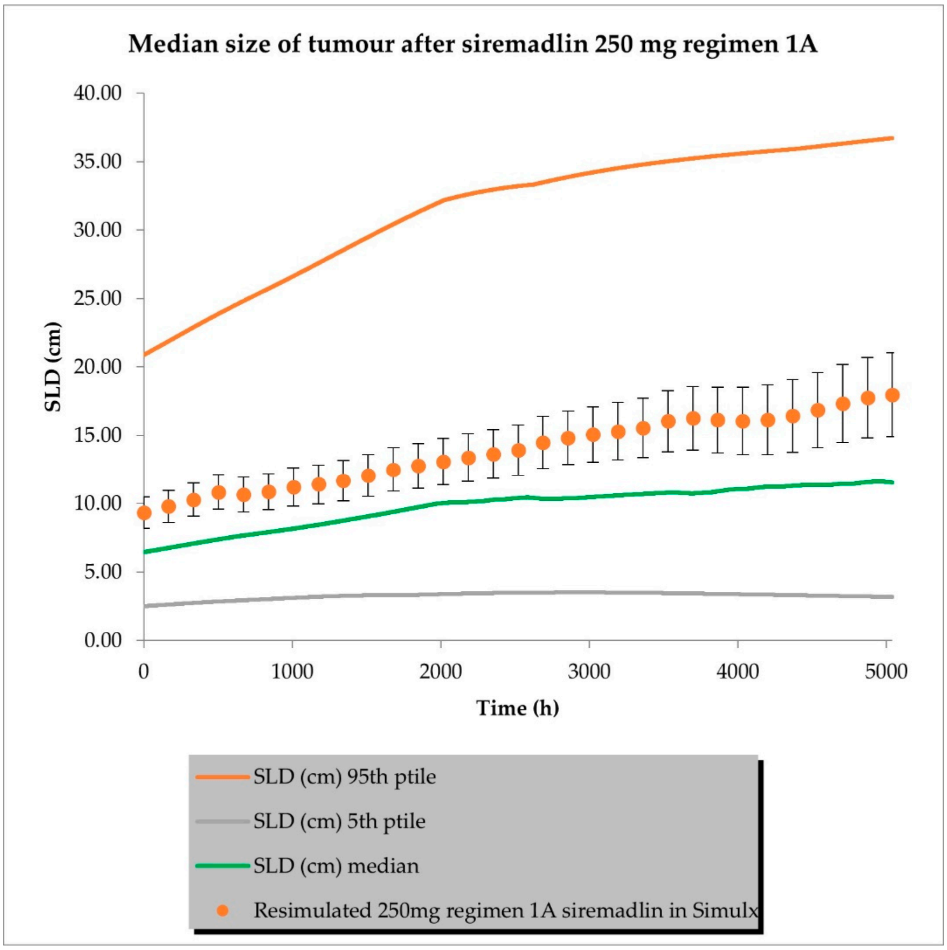

Figure S38. TGI model of 250 mg dose siremadlin administered in intermittent 1A regimen in cancer patients population. Resimulated data is presented as median ± SE from number of study participants (n = 9) n × 10.

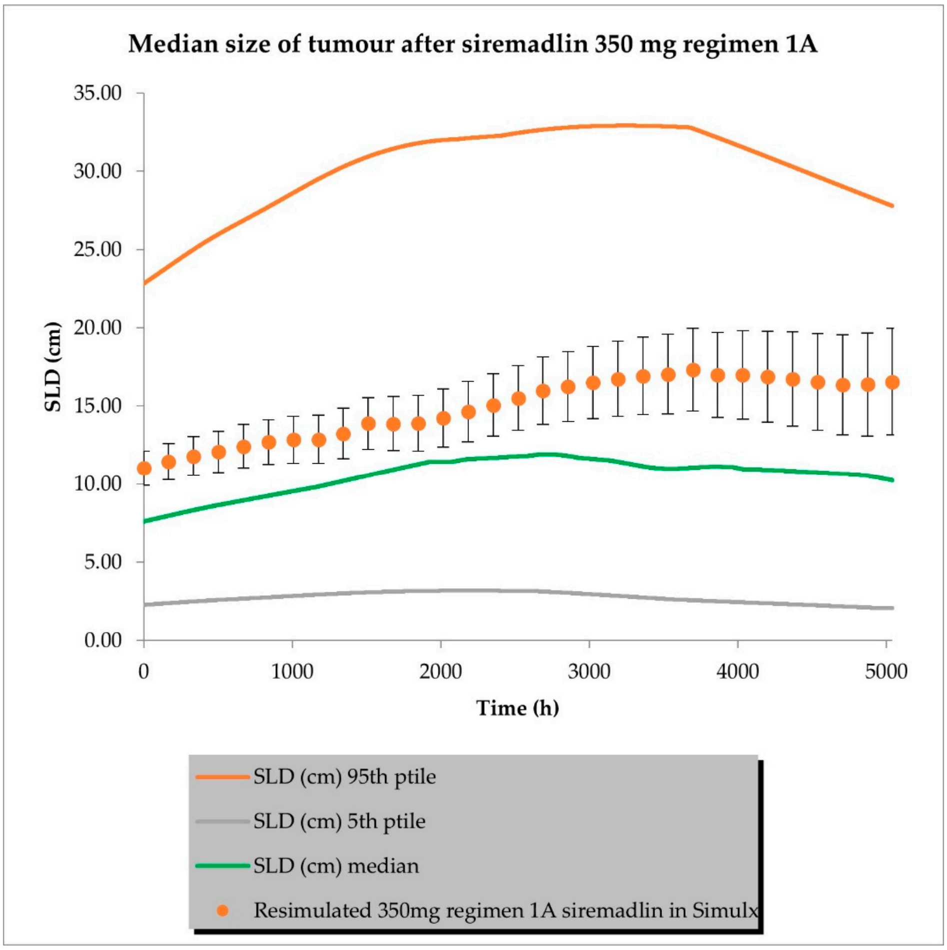

Figure S39. TGI model of 350 mg dose siremadlin administered in intermittent 1A regimen in cancer patients population. Resimulated data is presented as median ± SE from number of study participants (n = 5) n × 10.

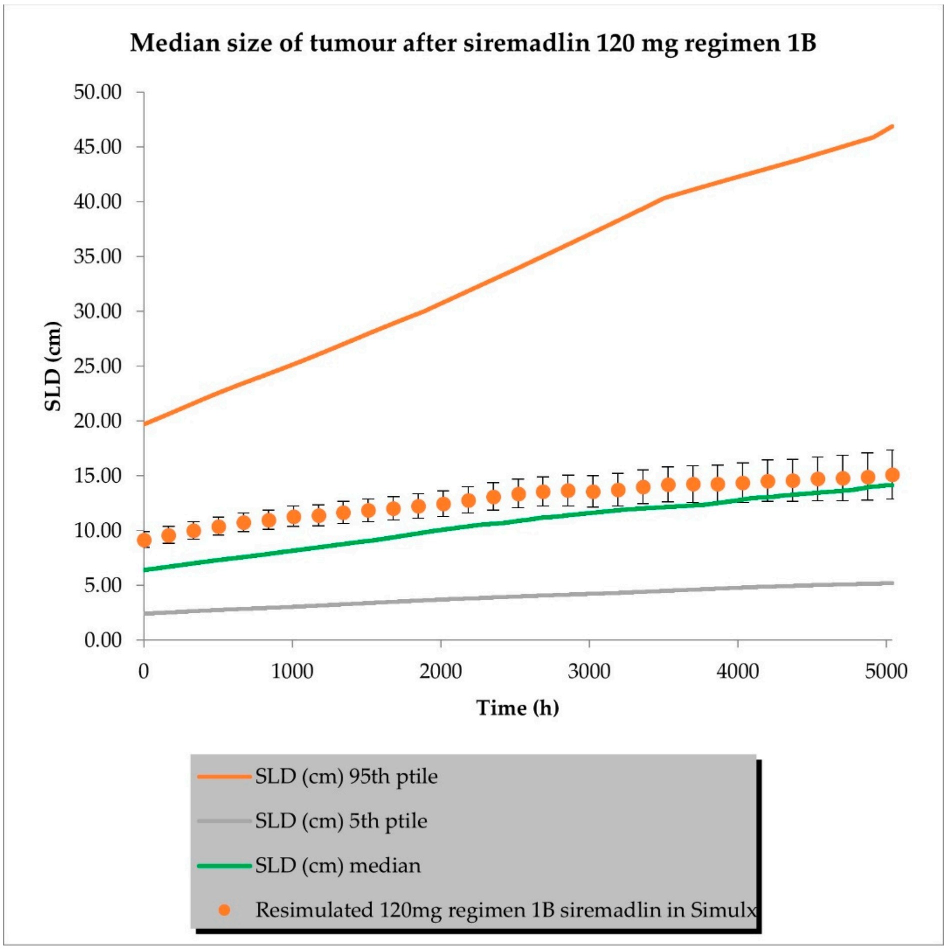

Figure S40. TGI model of 120 mg dose siremadlin administered in intermittent 1B regimen in cancer patients population. Resimulated data is presented as median ± SE from number of study participants (n = 29) n × 10.

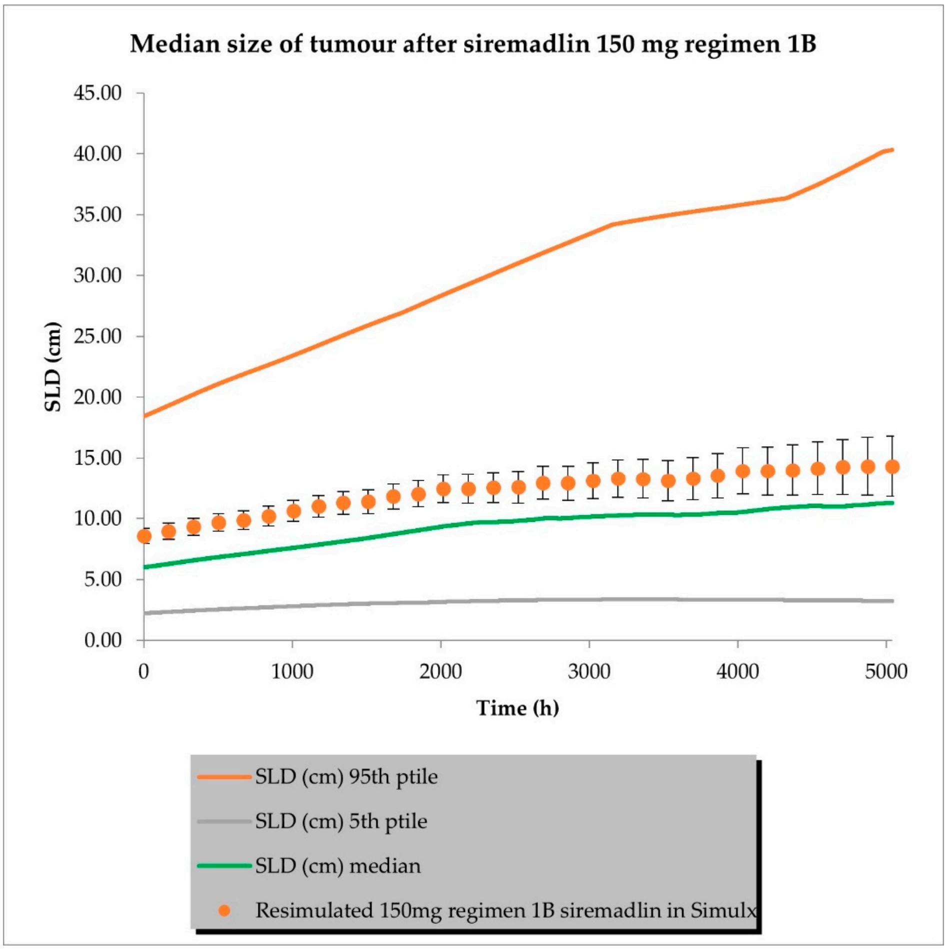

Figure S41. TGI model of 150 mg dose siremadlin administered in intermittent 1B regimen in cancer patients population. Resimulated data is presented as median ± SE from number of study participants (n = 15) n × 10.

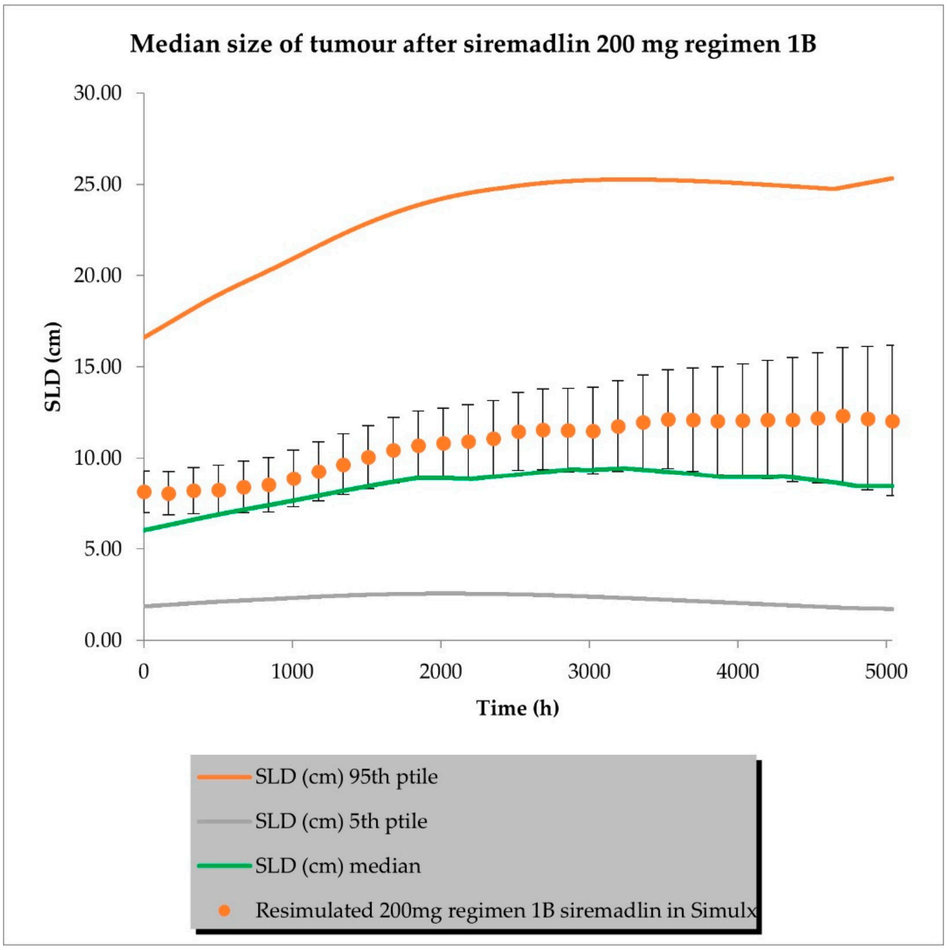

Figure S42. TGI model of 200 mg dose siremadlin administered in intermittent 1B regimen in cancer patients population. Resimulated data is presented as median ± SE from number of study participants (n = 3) n × 10.

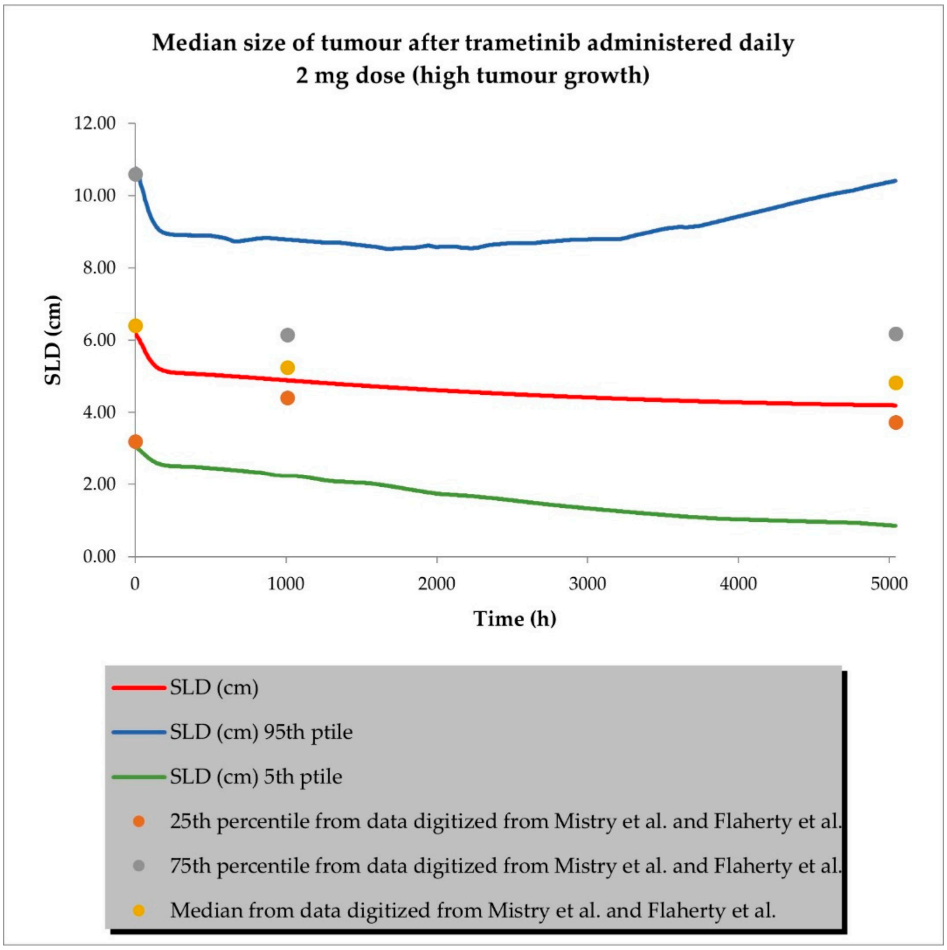

Figure S43. TGI model of trametinib administered in daily in cancer patient population (n = 214) with assumption of high tumour growth (kgh = 0.00028 1/h). Observed data presented as median from literature data (data digitized from Mistry et al. [22] and Flaherty et al. [13]).

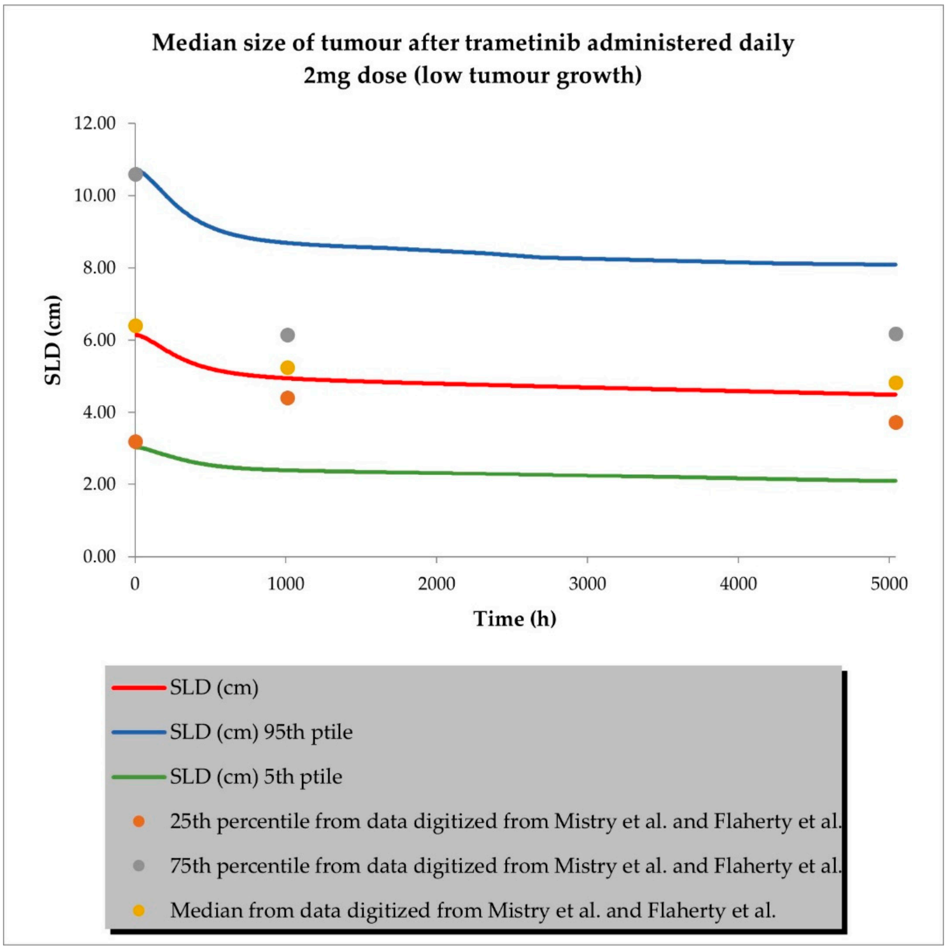

Figure S44. TGI model of trametinib administered in daily in cancer patient population (n = 214) with assumption of low tumour growth (kgh = 0.0000261 1/h). Observed data presented as median from literature data (data digitized from Mistry et al. [22] and Flaherty et al. [13]).

Code S1. Mlxtran code for resimulation of siremadlin pharmacokinetics and pharmacodynamics in Simulx.

```
[INDIVIDUAL]
input = {CL_pop, omega_CL, F1_pop, omega_F1, SLD0_pop, omega_SLD0, TSCs_pop, omega_TSCs, Tk01_pop, omega_Tk01, Tlag1_pop, omega_Tlag1, Tlag2_pop, omega_Tlag2, V_pop, omega_V, fs_pop, omega_fs, ka2_pop, omega_ka2, kgh_pop, omega_kgh, lambda_pop, omega_lambda, tau_pop, omega_tau}

DEFINITION:
CL = {distribution=logNormal, typical=CL_pop, sd=omega_CL}
F1 = {distribution=logitNormal, typical=F1_pop, sd=omega_F1}
SLD0 = {distribution=logNormal, typical=SLD0_pop, sd=omega_SLD0}
TSCs = {distribution=logNormal, typical=TSCs_pop, sd=omega_TSCs}
Tk01 = {distribution=logNormal, typical=Tk01_pop, sd=omega_Tk01}
Tlag1 = {distribution=logNormal, typical=Tlag1_pop, sd=omega_Tlag1}
Tlag2 = {distribution=logNormal, typical=Tlag2_pop, sd=omega_Tlag2}
V = {distribution=logNormal, typical=V_pop, sd=omega_V}
fs = {distribution=logitNormal, typical=fs_pop, sd=omega_fs}
ka2 = {distribution=logNormal, typical=ka2_pop, sd=omega_ka2}
```

kgh = {distribution=logNormal, typical=kgh_pop, sd=omega_kgh}
lambda = {distribution=logNormal, typical=lambda_pop, sd=omega_lambda}
tau = {distribution=logNormal, typical=tau_pop, sd=omega_tau}

[LONGITUDINAL]
input = {a1_PK, b1_PK, a1_PK_, b1_PK_}

DESCRIPTION:siremadlin Clinical TGI model.

Model for TS (tumour size):
 - Tumor growth follows an exponential model with exponential growth rate kgh and initial tumour size SLD0.

Tumor growth inhibition model:
 - Tumor growth inhibition modeled using a log-kill killing hypothesis where the treatment effect is linearly dependent on the drug exposure (TSCs*EXPOSURE).
 - A delay in treatment effect has been added by the introduction of 4 signal transit compartments (S1, S2, S3, S4). The length of this delay is determined by the parameter tau.
 - Model assumes emergence of a treatment-resistant tumour cell population which has a decreased sensitivity to the treatment (TCSr = TCSs/lambda). fs represents the proportion of the cells initially found within the tumour which belong to this treatment-sensitive cell population.

Treatment:
The treatment effect is based on EXPOSURE, which is the pharmacokinetics of the treatment modeled using the pkmodel macro.

Initial integration time is not fixed and is therefore the first dose or observation time for each subject. It can be fixed with "t_0 = ..." in the section EQUATION.
input = {Tk01, ka2, F1, Tlag1, Tlag2, V, CL, SLD0, kgh, fs, TSCs, lambda, tau}

PK:
k = CL/V

compartment(cmt = 1, volume = V, concentration = Cc)
absorption(cmt = 1, Tk0 = Tk01, Tlag = Tlag1, p = F1)
absorption(cmt = 1, ka = ka2, Tlag = Tlag2, p = 1-F1)
elimination(cmt = 1, k)

EXPOSURE = Cc

EQUATION:
odeType=stiff

```
;EXPOSURE = C_HDM

;initial conditions:
;lambda = TSCr/TSCs

Ns_0 = SLD0*fs
Nr_0 = SLD0*(1-fs)

S1_0 = 0
S2_0 = 0
S3_0 = 0
S4_0 = 0
; Signal distribution
;k2 = kgh/TSCs
AS = (kgh/TSCs*EXPOSURE)
ddt_S1 = (AS-S1)/tau
ddt_S2 = (S1-S2)/tau
ddt_S3 = (S2-S3)/tau
ddt_S4 = (S3-S4)/tau

TotalSLD = Ns+Nr

;Saturation for Ns and Nr at 1e12 to avoid infinite values
if Ns>1e12 | Nr>1e12
  NsDynamics = 0
  NrDynamics = 0
else
  NsDynamics = (kgh*Ns)-(S4*Ns)
  NrDynamics = (kgh*Nr)-(S4/(1+lambda)*Nr)
end
ddt_Ns = NsDynamics ; Treatment-sensitive cell population
ddt_Nr = NrDynamics ; Treatment-resistant cell population
;ddt_SLD = Ns+Nr
OUTPUT:
output = {TotalSLD, Cc}


DEFINITION:
y2_PD = {distribution=normal, prediction=TotalSLD, errorModel=combined1(a1_PK, b1_PK)}
y1_PK = {distribution=normal, prediction=Cc, errorModel=combined1(a1_PK_, b1_PK_)}
```

Code S2. Lua code for administration of siremadlin with mixed zero- and first-order absorption model.

```
function popSimSetup(...)
    sc:setNUserOdes(2) – number of differential equations
end

function odeInitStep(xin, su, P, ...)
su[1] = 0 -- for Substrate delay compartment (Zero Order absorption)
su[2] = 0 -- for Substrate delay compartment (First Order absorption)
return 0
end

function compoundSetup(...)
    --parameters names
   sc:setParameterName(1, "Tk0")
    sc:setParameterName(2, "kaSub")
    sc:setParameterName(3, "tlag1")
    sc:setParameterName(4, "tlag2")
    sc:setParameterName(5, "r")
    sc:setParameterName(6, "faSub")
    sc:setParameterName(7, "BPsub")
    sc:setParameterName(8, "vLiv")

    --IIV for siremadlin (CV derived from equation: ((e^(ω^2))-1)^1/2
   sc:setIIVDistribution(1, sc.LOGNORMAL_CV, 1.11, 0.0701)        --Tk0
   --sc:setIIVDistribution(1, sc.LOGNORMAL_CV, 0.11, 0.0701)      --Tk0 PK DDI
   sc:setIIVDistribution(2, sc.LOGNORMAL_CV, 1.00, 2.2776)        --kaSub
   --sc:setIIVDistribution(2, sc.LOGNORMAL_CV, 2.51, 2.2776)      --kaSub PK DDI
   sc:setIIVDistribution(3, sc.LOGNORMAL_CV, 0.688, 0.0500)       --tlag1
   sc:setIIVDistribution(4, sc.LOGNORMAL_CV, 0.41, 0.0200)        --tlag2
   sc:setIIVDistribution(5, sc.UNIFORM_MIN_MAX, 0.753, 0.0400)--r
   sc:setIIVDistribution(6, sc.LOGNORMAL_CV, 1.00, 0.3)           --faSub

end

function individualSetup(...)
    vLiv = 0.722*(sc:getIndivBSA())^1.176 -- liver volume for each patient in litres
   sc:setParameter(8, vLiv)
end


function odeRateStep(t, xin, su, gu, P, ...)

--Substrate (siremadlin) absorption parameters

local Tk0  =   sc:sampleIIVDistribution(1)
local kaSub  = sc:sampleIIVDistribution(2)
local tlag1  = sc:sampleIIVDistribution(3)
local tlag2  = sc:sampleIIVDistribution(4)
local r  =      sc:sampleIIVDistribution(5)
local faSub  =sc:sampleIIVDistribution(6)
local BPsub =  0.61

sc:setParameter(1, Tk0)
sc:setParameter(2, kaSub)
sc:setParameter(3, tlag1)
```

```
sc:setParameter(4, tlag2)
sc:setParameter(5, r)
sc:setParameter(6, faSub)


Tk0 =      P[1]
kaSub =  P[2]
tlag1 =   P[3]
tlag2 =   P[4]
r =         P[5]
faSub =  P[6]
BPsub =  P[7]

--Doses of siremadlin in uM
--local doseSub = 1.80047172359    --siremadlin 1mg dose
--local doseSub = 3.60094344718    --siremadlin 2mg dose
--local doseSub = 7.20188689437    --siremadlin 4mg dose
--local doseSub = 13.5035379269    --siremadlin 7.5mg dose
--local doseSub = 22.5058965449    --siremadlin 12.5mg dose
--local doseSub = 27.0070758539    --siremadlin 15mg dose
--local doseSub = 36.0094344718    --siremadlin 20mg dose
--local doseSub = 45.0117930898    --siremadlin 25mg dose
--local doseSub = 90.0235861796    --siremadlin 50mg dose
--local doseSub = 180.047172359    --siremadlin 100mg dose
local doseSub = 216.056606831    --siremadlin 120mg dose
--local doseSub = 270.070758538    --siremadlin 150mg dose
--local doseSub = 360.094344718    --siremadlin 200mg dose
--local doseSub = 450.117930898    --siremadlin 250mg dose
--local doseSub = 630.165103257    --siremadlin 350mg dose

--Dosing times
--local dosingtimesHDM = {0, 504, 1008, 1512, 2016, 2520, 3024, 3528, 4032, 4536, 5040} --qdx1/21day cycle (Reg1A)
local dosingtimesHDM = {0,168, 672, 840, 1344, 1512, 2016, 2184, 2688, 2856, 3360, 3528, 4032, 4200, 4704, 4872} --
qwx2/28day cycle (Reg1B)
--local dosingtimesHDM = {0, 24, 48, 72, 96, 120, 144,   168, 192, 216, 240,   264, 288, 312, 672,   696, 720, 744,
768,   792, 816, 840, 864,   888, 912, 936, 960,   984, 1344, 1368, 1392, 1416, 1440, 1464, 1488, 1512, 1536, 1560, 1584,
1608, 1632, 1656, 2016, 2040, 2064,   2088, 2112,   2136, 2160,   2184, 2208,   2232, 2256,   2280, 2304,   2328,
2688,   2712,   2736,   2760,   2784,   2808,   2832,   2856,   2880,   2904,   2928,   2952,   2976,   3000,   3360,   3384,
3408,   3432,   3456,   3480,   3504,   3528,   3552,   3576,   3600,   3624,   3648,   3672,   4032,   4056,   4080,   4104,
4128,   4152,   4176,   4200,   4224,   4248,   4272,   4296,   4320,   4344,   4704,   4728,   4752,   4776,   4800,   4824,
4848,   4872, 4896,   4920, 4944,   4968, 4992,   5016} --qdx14/28day cycle (Reg2A)
--local dosingtimesHDM = {0, 24, 48, 72, 96, 120, 144,   672, 696, 720, 744, 768, 792, 816, 1344, 1368, 1392, 1416, 1440,
1464, 1488, 2016, 2040, 2064,   2088, 2112,   2136, 2160,   2688, 2712,   2736, 2760,   2784, 2808,   2832, 3360,   3384,
3408,   3432,   3456,   3480,   3504,   4032,   4056,   4080,   4104,   4128,   4152,   4176,   4704,   4728,   4752,   4776,
4800,   4824, 4848} --qdx7/28day cycle (Reg2C)


--for i=1,11  --qdx1/21day cycle (Reg1A)
--for i=1,112 --qdx14/28day cycle (Reg2A)
--for i=1,56  --qdx7/28day cycle (Reg2C)
for i=1,16  --qwx2/28day cycle (Reg1B)
do
if (t>=dosingtimesHDM[i]+tlag1) and (t<=dosingtimesHDM[i]+(Tk0+tlag1))
then
```

```
su[1] = doseSub*r/Tk0 --dose administerad via zero order absorption
end

if (t>=dosingtimesHDM[i]+tlag2) and (t<=dosingtimesHDM[i]+(Tk0+tlag1))
then
su[2] = kaSub*doseSub*(1-r) --dose administered via first order absorption
end
end

gu[1] = - su[1] -- zero order ODE
gu[2] = - su[2] -- first order ODE

SubAbsCompDelayZO = su[1]
SubAbsCompDelayFO = su[2]


local SubsysGradient = sc:getGradient(0) --for substrate (siremadlin)
local newSubsysGradient = SubsysGradient + (SubAbsCompDelayZO + SubAbsCompDelayFO)*BPsub/vLiv*faSub
sc:setGradient(0,newSubsysGradient)

   return SubAbsCompDelayZO, SubAbsCompDelayFO
end
```

Code S3. Lua code for administration of trametinib in drug interaction model (siremadlin + trametinib combination).

```
function popSimSetup(...)
      return 0
end

function odeInitStep(xin, su, P, ...)
      return 0
end

function individualSetup(...)
      vLiv = 0.722*(sc:getIndivBSA())^1.176 -- liver volume for each patient in litres
   sc:setParameter(4, vLiv)
end

function odeRateStep(t, xin, su, gu, P, ...)

--Inhibitor (trametinib) absorption parameters without PK interaction (PK DDI)
local faInh = 0.72 -- fraction absorbed
local kaInh = 0.6 -- absorption rate constant
local tlagInh = 0.35 -- lag time
local BPInh =  0.56 -- blood to plasma ratio


--Coadministration times
--local Coadministrationtimes = {0, 504, 1008, 1512, 2016, 2520, 3024, 3528, 4032, 4536, 5040} --coadministration times with Reg1A
local Coadministrationtimes = {0,168, 672, 840, 1344, 1512, 2016, 2184, 2688, 2856, 3360, 3528, 4032, 4200, 4704, 4872} --coadministration times with Reg1B
--local Coadministrationtimes = {0, 24, 48, 72, 96, 120, 144,    168, 192, 216, 240,    264, 288, 312, 672,    696, 720, 744,
```

```
768, 792, 816, 840, 864, 888, 912, 936, 960, 984, 1344, 1368, 1392, 1416, 1440, 1464, 1488, 1512, 1536, 1560, 1584, 1608, 1632, 1656, 2016, 2040, 2064, 2088, 2112, 2136, 2160, 2184, 2208, 2232, 2256, 2280, 2304, 2328, 2688, 2712, 2736, 2760, 2784, 2808, 2832, 2856, 2880, 2904, 2928, 2952, 2976, 3000, 3360, 3384, 3408, 3432, 3456, 3480, 3504, 3528, 3552, 3576, 3600, 3624, 3648, 3672, 4032, 4056, 4080, 4104, 4128, 4152, 4176, 4200, 4224, 4248, 4272, 4296, 4320, 4344, 4704, 4728, 4752, 4776, 4800, 4824, 4848, 4872, 4896, 4920, 4944, 4968, 4992, 5016} --coadministration times with Reg2A
--local Coadministrationtimes = {0, 24, 48, 72, 96, 120, 144, 672, 696, 720, 744, 768, 792, 816, 1344, 1368, 1392, 1416, 1440, 1464, 1488, 2016, 2040, 2064, 2088, 2112, 2136, 2160, 2688, 2712, 2736, 2760, 2784, 2808, 2832, 3360, 3384, 3408, 3432, 3456, 3480, 3504, 4032, 4056, 4080, 4104, 4128, 4152, 4176, 4704, 4728, 4752, 4776, 4800, 4824, 4848} --coadministration times with Reg2C


--for i=1,11  --for coadministration times with Reg1A
--for i=1,112 --for coadministration times with Reg2A
--for i=1,56  --for coadministration times with Reg2C
for i=1,16  --for coadministration times with Reg1B
do
if (t>=Coadministrationtimes[i] and t<=(Coadministrationtimes[i]+24))
then
   --Inhibitor (trametinib) parameters with PK interaction (PK DDI)
   faInh = 0.608 -- fraction absorbed
   kaInh = 0.252 -- absorption rate constant
   tlagInh = 0 -- lag time
end
end

local doseInh = 3.24997156275 -- dose of trametinib 2 mg in uM
local RateInInh = 0

--Dosing times
for j=0,5040,24 -- daily administration over simulation timeframe
do
if (t>=(j+tlagInh))
then
RateInInh = RateInInh + doseInh*kaInh*faInh*math.exp(- kaInh * (t-tlagInh-j)) --first order absorption of trametinib
end
end


sc:setParameter(1, faInh)
sc:setParameter(2, kaInh)
sc:setParameter(3, tlagInh)


faInh =     P[1]
kaInh =     P[2]
tlagInh =   P[3]


local InhsysGradient = sc:getGradient(21) --for inhibitor (trametinib)
local newInhsysGradient = InhsysGradient + RateInInh/vLiv
sc:setGradient(21,newInhsysGradient)
```

```
  return RateInInh
end
```

Code S4. Lua code for tumour growth inhibition model for siremadlin + trametinib combination 120 + 2 mg administered in regimen 1B for siremadlin and daily for trametinib.

```
--Drug Combination Custom PD TGI model features:
--Tumor growth follows logistic model
--Killing hypothesis: Log-kill killing hypothesis with exponential kill kinetics
--Delay in treatment effect (Signal distribution model of delay - Lobo & Balthasar 2002) using 4 transit compartments
--Modeling of acquired resistance (2 subpopulations: sensitive and resistant)
--Individual setup and lognormal distribution for parameters
--parameters estimates taken from Monolix model

function popSimSetup(...)
                    --insert user code
            --set parameters name (not all parameters will be used - not all kkill values, stored for sript modification for drug combinations)
            sc:setNUserOdes(6)    --number of differential equations (number of "gu"s)
            sc:setParameterName(1, "SLD0")            --initial tumour size
            sc:setParameterName(2, "kgh")             --tumour growth
            sc:setParameterName(3, "fs")              --sensitive cells fraction
            sc:setParameterName(4, "lambda")          --ressistance factor (kkillr/kkill)
            sc:setParameterName(5, "tau")             --killing effect delay
            sc:setParameterName(6, "TSCs")            --killing constant for sensitive population of cancer cells
            sc:setParameterName(7, "Ts0")             --initial volume of sensitive population of cancer cells
            sc:setParameterName(8, "Tsr0")            --initial volume of resistant population of cancer cells
            sc:setParameterName(9, "TSCs_HDM")     --siremadlin killing constant for sensitive poupation of cancer cells
            sc:setParameterName(10, "TSCs_TRA")     --trametinib killing constant for sensitive poupation of cancer cells
            sc:setParameterName(11, "gamma")        --PD interaction parameter (beta parameter) from in vitro studies
            sc:setParameterName(12, "AUCratioHDM201")    --siremadlin exposure ratio predicted from animal model
            sc:setParameterName(13, "AUCratiotrametinib") --trametinib exposure ratio predicted from animal model

  --IIV for siremadlin and trametinib (CV derived from equation: ((e^(ω^2))-1)^1/2
  --sc:setIIVDistribution(2, sc.LOGNORMAL_CV, 0.0000261, 0.306878288)   --kgh low [1/h]
  sc:setIIVDistribution(2, sc.LOGNORMAL_CV, 0.00028, 0.306878288)        --kgh high [1/h]
  sc:setIIVDistribution(3, sc.LOGNORMAL_CV, 0.0321, 6.361374524)         --fs HDM [%]
  --sc:setIIVDistribution(3, sc.LOGNORMAL_CV, 0.061953, 6.361374524)     --fs low [%]
  sc:setIIVDistribution(4, sc.LOGNORMAL_CV, 132, 0.100250522)            --lambda HDM [unitless]
  sc:setIIVDistribution(5, sc.LOGNORMAL_CV, 558, 1.005748851)            --tau HDM [h]
  sc:setIIVDistribution(6, sc.LOGNORMAL_CV, 1.01546605, 0.100250522)     --TSCs HDM [nM]
  sc:setIIVDistribution(9, sc.LOGNORMAL_CV, 0.191, 1)                    --fs TRA [%]
  sc:setIIVDistribution(10, sc.LOGNORMAL_CV, 94.3, 0.1)                  --lambda TRA [unitless]
  sc:setIIVDistribution(11, sc.LOGNORMAL_CV, 2.5, 0.1)                   --tau TRA [h]
  sc:setIIVDistribution(12, sc.LOGNORMAL_CV, 0.258, 0.1)                 --TSCs TRA (kgh high) [nM]
  --sc:setIIVDistribution(12, sc.LOGNORMAL_CV, 0.177, 0.1)               --TSCs TRA (kgh low) [nM]
  sc:setIIVDistribution(13, sc.NORMAL_SD, 1.2312, 0.048) -- beta PD interaction parameter from in vitro studies
end
```

```
function individualSetup(...)
      local SLD0  = sc:getIndivInitTumourVol() --initial tumour size
   local kgh  = sc:sampleIIVDistribution(2)    --tumour growth
   local fs  = sc:sampleIIVDistribution(3)+sc:sampleIIVDistribution(9)                    --sensitive cells fraction
   local lambda  = sc:sampleIIVDistribution(4)           --ressistance factor (kkillr/kkill)
   local tau  = sc:sampleIIVDistribution(5)-sc:sampleIIVDistribution(11)                  --killing effect delay
   local TSCs_HDM = sc:sampleIIVDistribution(6)                  --killing constant for sensitive poupation of cancer
cells
   local TSCs_TRA = sc:sampleIIVDistribution(12)                 --killing constant for sensitive poupation of cancer
cells
   local TSCs = TSCs_TRA

      sc:setParameter(1, SLD0)
      sc:setParameter(2, kgh)
      sc:setParameter(3, fs)
      sc:setParameter(4, lambda)
      sc:setParameter(5, tau)
      sc:setParameter(6, TSCs)
      sc:setParameter(9,  TSCs_HDM)
      sc:setParameter(10, TSCs_TRA)

      Ts0 = SLD0*fs        --initial size of sensitive population of cancer cells
   Tsr0 = SLD0*(1-fs)    --initial size of resistant population of cancer cells

   sc:setParameter(7, Ts0)
      sc:setParameter(8, Tsr0)

      local AUCratioHDM201 = 1.8240
   local AUCratiotrametinib = 1.2438
   local gamma = sc:sampleIIVDistribution(13)

      sc:setParameter(11, gamma)
      sc:setParameter(12, AUCratioHDM201)
   sc:setParameter(13, AUCratiotrametinib)
end

function odeInitStep(su, P, ...)

   -- delay transit compartments
   su[1] = 0       --  K1 transit copartments for sensitive population
   su[2] = 0       --  K2
   su[3] = 0       --  K3
   su[4] = 0       --  K4
   su[5] = P[7]   -- TS estimate
   su[6] = P[8]   -- TSr estimate
   return 0

end



function odeRateStep(t,su,gu,P,...)
```

```
Ca = sc:getIndivPlasmaConc(sc.SUBSTRATE)    -- first drug: siremadlin units: uM
Cb = sc:getIndivPlasmaConc(sc.INH1)    -- second drug: trametinib units: uM

C_HDM = 1000 * Ca
  C_TRA =   1000 * Cb

  local SLD0, kgh, fs, lambda, tau, TSCs, Ts0, Tsr0, TSCs_HDM, TSCs_TRA, gamma, AUCratioHDM201, AUCratiotrametinib

SLD0  =              P[1]
  kgh  =                   P[2]
  fs  =                    P[3]
  lambda  =                P[4]
  tau  =                   P[5]
  TSCs =                   P[6]
  Ts0 =                    P[7]
  Tsr0 =                   P[8]
  TSCs_HDM =               P[9]
  TSCs_TRA =               P[10]
  gamma =                  P[11]
  AUCratioHDM201 =         P[12]
  AUCratiotrametinib =     P[13]


  --Coadministration times
--local Coadministrationtimes = {0, 504, 1008, 1512, 2016, 2520, 3024, 3528, 4032, 4536, 5040} --coadministration times with Reg1A
local Coadministrationtimes = {0,168, 672, 840, 1344, 1512, 2016, 2184, 2688, 2856, 3360, 3528, 4032, 4200, 4704, 4872} --coadministration times with Reg1B
--local Coadministrationtimes = {0, 24, 48, 72, 96, 120, 144,   168, 192, 216, 240,   264, 288, 312, 672,   696, 720, 744, 768,   792, 816, 840, 864,   888, 912, 936, 960,   984, 1344, 1368, 1392, 1416, 1440, 1464, 1488, 1512, 1536, 1560, 1584, 1608, 1632, 1656, 2016, 2040, 2064,   2088, 2112,   2136, 2160,   2184, 2208,   2232, 2256,   2280, 2304,   2328, 2688,   2712,   2736,   2760,   2784,   2808,   2832,   2856,   2880,   2904,   2928,   2952,   2976,   3000,   3360,   3384, 3408,   3432,   3456,   3480,   3504,   3528,   3552,   3576,   3600,   3624,   3648,   3672,   4032,   4056,   4080,   4104, 4128,   4152,   4176,   4200,   4224,   4248,   4272,   4296,   4320,   4344,   4704,   4728,   4752,   4776,   4800,   4824, 4848,   4872, 4896,   4920, 4944,   4968, 4992,   5016} --coadministration times with Reg2A
--local Coadministrationtimes = {0, 24, 48, 72, 96, 120, 144,   672, 696, 720, 744, 768, 792, 816, 1344, 1368, 1392, 1416, 1440, 1464, 1488, 2016, 2040, 2064,   2088, 2112,   2136, 2160,   2688, 2712,   2736, 2760,   2784, 2808,   2832, 3360,   3384,   3408,   3432,   3456,   3480,   3504,   4032,   4056,   4080,   4104,   4128,   4152,   4176,   4704,   4728, 4752,   4776, 4800,   4824, 4848} --coadministration times with Reg2C


--for i=1,11  --for coadministration times with Reg1A
--for i=1,112 --for coadministration times with Reg2A
--for i=1,56  --for coadministration times with Reg2C
for i=1,16  --for coadministration times with Reg1B
do
if (t>=Coadministrationtimes[i] and t<=(Coadministrationtimes[i]+24))
  then
   TSCs = TSCs_HDM - TSCs_TRA --no PK/PD DDI simple additive effect (Scenario 1)
   --TSCs = (TSCs_HDM - TSCs_TRA)/gamma --PD DDI synergistic effect (Scenario 2)
   --TSCs = (TSCs_HDM/AUCratioHDM201 - TSCs_TRA/AUCratiotrametinib) --PK DDI synergistic effect (Scenario 3)
```

```
  --TSCs = (TSCs_HDM/AUCratioHDM201 - TSCs_TRA/AUCratiotrametinib)/gamma --PK/PD DDI synergistic effect (Scenario 4)
 end
 end

 --Delay of effect for sensitive cancer population

 local TK = kgh/TSCs*(C_HDM+C_TRA)

   gu[1] = (TK -su[1])    / tau
   gu[2] = (su[1] - su[2]) / tau
   gu[3] = (su[2] - su[3]) / tau
   gu[4] = (su[3] - su[4]) / tau

   TTS = su[5] + su[6] --Total tumour size (TTS)

 if (su[5] > 1E12) or (su[6] > 1E12) then
   gu[5] = 0
   gu[6] = 0
 else
   gu[5] = (kgh*su[5]) - (su[4]*su[5])
       gu[6] = (kgh*su[6]) - (su[4]/(1+lambda)*su[6])
   end

 TS =  su[5]
   TSr = su[6]

   TotalTumorSize = TS + TSr

    sc:feedbackTumourVol(TotalTumorSize)
   return TotalTumorSize
   end
```

References

1. Hofmann, F. Small Molecule HDM201 Inhibitor HDM201. Presented at the AACR Annual Meeting 2016, New Orleans, LA, USA, 16–20 April 2016.
2. Guerreiro, N.; Jullion, A.; Ferretti, S.; Fabre, C.; Meille, C. Translational Modeling of Anticancer Efficacy to Predict Clinical Outcomes in a First-in-Human Phase 1 Study of MDM2 Inhibitor HDM201. *AAPS J* **2021**, *23*, 28, doi:10.1208/s12248-020-00551-z.
3. Lei, Y.; Zhenglin, Y.; Heng, L. MDM2 Inhibitors. U.S. Patent 11,339,171, 24 May 2022.
4. Jeay, S.; Ferretti, S.; Holzer, P.; Fuchs, J.; Chapeau, E.A.; Wartmann, M.; Sterker, D.; Romanet, V.; Murakami, M.; Kerr, G.; et al. Dose and Schedule Determine Distinct Molecular Mechanisms Underlying the Efficacy of the P53-MDM2 Inhibitor HDM201. *Cancer Res* **2018**, *78*, 6257–6267, doi:10.1158/0008-5472.CAN-18-0338.
5. Witkowski, J.; Polak, S.; Rogulski, Z.; Pawelec, D. In Vitro/In Vivo Translation of Synergistic Combination of MDM2 and MEK Inhibitors in Melanoma Using PBPK/PD Modelling: Part II. *International Journal of Molecular Sciences* **2022**, *23*, 11939, doi:10.3390/ijms231911939.
6. Paine, S.W.; Ménochet, K.; Denton, R.; McGinnity, D.F.; Riley, R.J. Prediction of Human Renal Clearance from Preclinical Species for a Diverse Set of Drugs That Exhibit Both Active Secretion and Net Reabsorption. *Drug Metab Dispos* **2011**, *39*, 1008–1013, doi:10.1124/dmd.110.037267.

7. Stein, E.M.; DeAngelo, D.J.; Chromik, J.; Chatterjee, M.; Bauer, S.; Lin, C.-C.; Suarez, C.; de Vos, F.; Steeghs, N.; Cassier, P.A.; et al. Results from a First-in-Human Phase I Study of Siremadlin (HDM201) in Patients with Advanced Wild-Type TP53 Solid Tumors and Acute Leukemia. *Clin Cancer Res* **2022**, *28*, 870–881, doi:10.1158/1078-0432.CCR-21-1295.
8. Food and Drug Administration (FDA), Trametinib Pharmacology Review. Available online: https://www.accessdata.fda.gov/drugsatfda_docs/nda/2013/204114Orig1s000PharmR.pdf (accessed on 15 December 2022).
9. Leonowens, C.; Pendry, C.; Bauman, J.; Young, G.C.; Ho, M.; Henriquez, F.; Fang, L.; Morrison, R.A.; Orford, K.; Ouellet, D. Concomitant Oral and Intravenous Pharmacokinetics of Trametinib, a MEK Inhibitor, in Subjects with Solid Tumours. *British Journal of Clinical Pharmacology* **2014**, *78*, 524–532, doi:10.1111/bcp.12373.
10. Kallinowski, F.; Schlenger, K.H.; Runkel, S.; Kloes, M.; Stohrer, M.; Okunieff, P.; Vaupel, P. Blood Flow, Metabolism, Cellular Microenvironment, and Growth Rate of Human Tumor Xenografts. *Cancer Res* **1989**, *49*, 3759–3764.
11. Benjaminsen, I.C.; Graff, B.A.; Brurberg, K.G.; Rofstad, E.K. Assessment of Tumor Blood Perfusion by High-Resolution Dynamic Contrast-Enhanced MRI: A Preclinical Study of Human Melanoma Xenografts. *Magn Reson Med* **2004**, *52*, 269–276, doi:10.1002/mrm.20149.
12. Graff, B.A.; Benjaminsen, I.C.; Melås, E.A.; Brurberg, K.G.; Rofstad, E.K. Changes in Intratumor Heterogeneity in Blood Perfusion in Intradermal Human Melanoma Xenografts during Tumor Growth Assessed by DCE-MRI. *Magn Reson Imaging* **2005**, *23*, 961–966, doi:10.1016/j.mri.2005.09.006.
13. Flaherty, K.T.; Robert, C.; Hersey, P.; Nathan, P.; Garbe, C.; Milhem, M.; Demidov, L.V.; Hassel, J.C.; Rutkowski, P.; Mohr, P.; et al. Improved Survival with MEK Inhibition in BRAF-Mutated Melanoma. *New England Journal of Medicine* **2012**, *367*, 107–114, doi:10.1056/NEJMoa1203421.
14. Elassaiss-Schaap, Jeroen; Heisterkamp, Siem Variability as Constant Coefficient of Variation: Can We Right Two Decades in Error? PAGE meeting (2009), Abstract 1508 Available online: https://www.page-meeting.org/pdf_assets/4964-Elassaiss-Schaap%20-%20Equations%20variability%20reporting%20PK-PD%20-%20Final.pdf (accessed on 15 December 2022)
15. Witkowski, J.; Polak, S.; Rogulski, Z.; Pawelec, D. In Vitro/In Vivo Translation of Synergistic Combination of MDM2 and MEK Inhibitors in Melanoma Using PBPK/PD Modelling: Part I. *International Journal of Molecular Sciences* **2022**, *23*, 12984, doi:10.3390/ijms232112984.
16. Koo, H.-M.; VanBrocklin, M.; McWilliams, M.J.; Leppla, S.H.; Duesbery, N.S.; Woude, G.F.V. Apoptosis and Melanogenesis in Human Melanoma Cells Induced by Anthrax Lethal Factor Inactivation of Mitogen-Activated Protein Kinase Kinase. *Proceedings of the National Academy of Sciences* **2002**, *99*, 3052–3057, doi:10.1073/pnas.052707699.
17. Alesiani, D.; Cicconi, R.; Mattei, M.; Bei, R.; Canini, A. Inhibition of Mek 1/2 Kinase Activity and Stimulation of Melanogenesis by 5,7-Dimethoxycoumarin Treatment of Melanoma Cells. *International Journal of Oncology* **2009**, *34*, 1727–1735, doi:10.3892/ijo_00000303.
18. Saud, A.; Sagineedu, S.R.; Ng, H.-S.; Stanslas, J.; Lim, J.C.W. Melanoma Metastasis: What Role Does Melanin Play? (Review). *Oncology Reports* **2022**, *48*, 1–10, doi:10.3892/or.2022.8432.
19. Sarna, M.; Krzykawska-Serda, M.; Jakubowska, M.; Zadlo, A.; Urbanska, K. Melanin Presence Inhibits Melanoma Cell Spread in Mice in a Unique Mechanical Fashion. *Sci Rep* **2019**, *9*, 9280, doi:10.1038/s41598-019-45643-9.

20. Wu, C.-E.; Koay, T.S.; Esfandiari, A.; Ho, Y.-H.; Lovat, P.; Lunec, J. ATM Dependent DUSP6 Modulation of P53 Involved in Synergistic Targeting of MAPK and P53 Pathways with Trametinib and MDM2 Inhibitors in Cutaneous Melanoma. *Cancers (Basel)* **2018**, *11*, 3, doi:10.3390/cancers11010003.
21. Infante, J.R.; Fecher, L.A.; Falchook, G.S.; Nallapareddy, S.; Gordon, M.S.; Becerra, C.; DeMarini, D.J.; Cox, D.S.; Xu, Y.; Morris, S.R.; et al. Safety, Pharmacokinetic, Pharmacodynamic, and Efficacy Data for the Oral MEK Inhibitor Trametinib: A Phase 1 Dose-Escalation Trial. *The Lancet Oncology* **2012**, *13*, 773–781, doi:10.1016/S1470-2045(12)70270-X.
22. Mistry, H.B.; Orrell, D.; Eftimie, R. Model Based Analysis of the Heterogeneity in the Tumour Size Dynamics Differentiates Vemurafenib, Dabrafenib and Trametinib in Metastatic Melanoma. *Cancer Chemother Pharmacol* **2018**, *81*, 325–332, doi:10.1007/s00280-017-3486-3.
